# Genomic and phenotypic characterization of a red-pigmented strain of *Massilia frigida* isolated from an Antarctic microbial mat

**DOI:** 10.3389/fmicb.2023.1156033

**Published:** 2023-05-12

**Authors:** Jacob M. C. Shaffer, Lesley-Ann Giddings, Robert M. Samples, Jill A. Mikucki

**Affiliations:** ^1^Department of Microbiology, University of Tennessee, Knoxville, TN, United States; ^2^Department of Chemistry, Smith College, Northampton, MA, United States

**Keywords:** prodigiosin biosynthesis, Antarctic *Massilia*, psychrophilic bacteria, whole-genome sequencing, Don Juan Pond Basin

## Abstract

The McMurdo Dry Valleys of Antarctica experience a range of selective pressures, including extreme seasonal variation in temperature, water and nutrient availability, and UV radiation. Microbial mats in this ecosystem harbor dense concentrations of biomass in an otherwise desolate environment. Microbial inhabitants must mitigate these selective pressures *via* specialized enzymes, changes to the cellular envelope, and the production of secondary metabolites, such as pigments and osmoprotectants. Here, we describe the isolation and characterization of a Gram-negative, rod-shaped, motile, red-pigmented bacterium, strain DJPM01, from a microbial mat within the Don Juan Pond Basin of Wright Valley. Analysis of strain DJMP01’s genome indicates it can be classified as a member of the *Massilia frigida* species. The genome contains several genes associated with cold and salt tolerance, including multiple RNA helicases, protein chaperones, and cation/proton antiporters. In addition, we identified 17 putative secondary metabolite gene clusters, including a number of nonribosomal peptides and ribosomally synthesized and post-translationally modified peptides (RiPPs), among others, and the biosynthesis pathway for the antimicrobial pigment prodigiosin. When cultivated on complex agar, multiple prodiginines, including the antibiotic prodigiosin, 2-methyl-3-propyl-prodiginine, 2-methyl-3-butyl-prodiginine, 2-methyl-3-heptyl-prodiginine, and cycloprodigiosin, were detected by LC–MS. Genome analyses of sequenced members of the *Massilia* genus indicates prodigiosin production is unique to Antarctic strains. UV-A radiation, an ecological stressor in the Antarctic, was found to significantly decrease the abundance of prodiginines produced by strain DJPM01. Genomic and phenotypic evidence indicates strain DJPM01 can respond to the ecological conditions of the DJP microbial mat, with prodiginines produced under a range of conditions, including extreme UV radiation.

## 1. Introduction

Upwards of 80% of Earth’s environments are permanently cold (<5°C), including polar, alpine, maritime, and atmospheric regions (De Maayer et al., 2014). Understanding how life survives at low temperatures is crucial to our understanding of life itself, as well as how life might exist on other planets. On the Antarctic continent, the McMurdo Dry Valleys (MDV) constitute the largest ice-free region (Levy, 2013). The MDV region is the coldest, driest desert on our planet, and experiences average annual air and absolute surficial soil temperatures ranging from −26.8 to −16.5°C and −59.8 to +31.1°C, respectively (Obryk et al., 2020), and receive a maximum of ~50 mm of precipitation in the form of snowfall annually (Fountain et al., 2009). Additionally, the MDV experiences long periods of sustained light and dark, due to its extreme southern latitude, and high levels of UV-A and UV-B radiation from thinning in the ozone layer (Frederick and Snell, 1988).

Antarctic microbial mats provide a unique system for studying the effects of UV radiation and other adaptations due to their high biomass compared to other microbial niches within the MDV (Davey and Clarke, 1992). The formation of these communities is reliant on photosynthetic cyanobacteria, including either *Nostoc* species or *Phormidium* and *Oscillatoria* species, depending on the geomorphology and hydrology of the niche (Alger et al., 1997). Other photosynthetic organisms, such as green algae and diatoms, can be found in Antarctic mats, as well as heterotrophic bacteria and microscopic invertebrates (Andriuzzi et al., 2018). Microbial mats that occupy perennial stream channels and shallow ponds are particularly exposed to extremes of seasonal variation and experience fluctuations in meltwater flow from glaciers due to changes in temperature and solar angle, which in turn can lead to changes in nutrient availability and community structure (Zoumplis et al., 2023). In the winter, communities are exposed to months of darkness, desiccation, and subzero temperatures. MDV mats in stream channels and surface ponds lie dormant for ~10 months of the year due to the lack of water and sunlight, followed by rapid reactivation in response to rehydration by glacial meltwater (McKnight et al., 2007). In addition to freezing and dehydration, MDV mats are exposed to intense ultraviolet (UV) radiation. This UV exposure causes DNA damage and the production of reactive oxygen species (ROS; Castenholz and Garcia-Pichel, 2012). UV radiation has been proposed as a major limiting factor on growth for communities within the MDV (Tosi et al., 2005).

**Figure 1:**
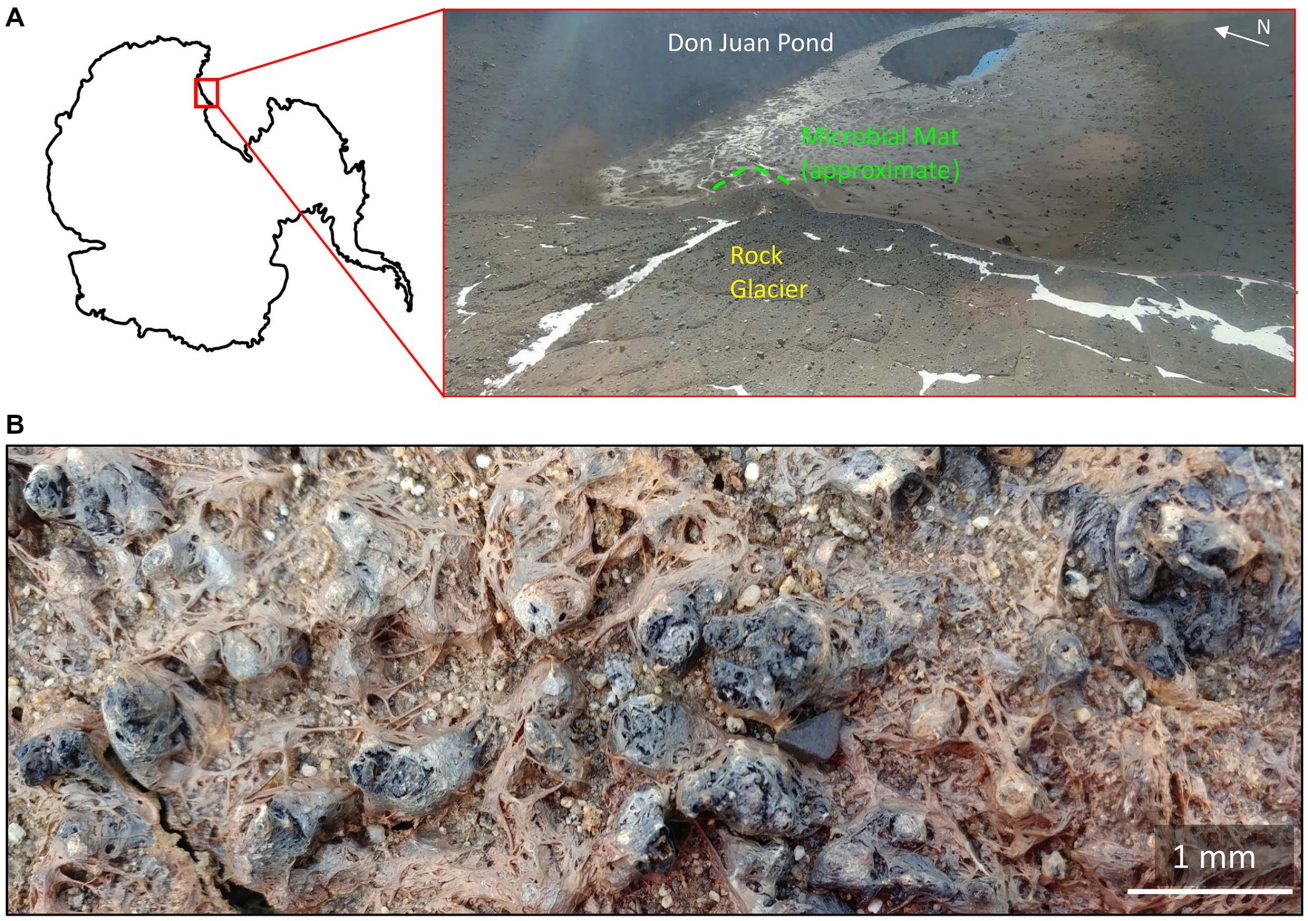
The Don Juan Pond (DJP) microbial mat **(A)** Location of the microbial mat within DJP basin. Approximate location of the basin in Antarctica is inset. **(B)** Close up of the DJP basin microbial mat collected for enrichment cultures that yielded strain DJPM01.

Microbial mats are scattered throughout the MDV, largely concentrated in meltwater streams and lake margins (Power et al., 2020) that wax and wane in volume in response to climate (Mikucki et al., 2010). Within the South Fork of the Wright Valley is a near-saturated CaCl_2_ brine pond, known as Don Juan Pond (DJP). DJP is bounded to the west by a rock glacier, which provides a source of meltwater to the basin (Figure 1). While there are conflicting reports regarding the habitability of the brine (Toner et al., 2017), the DJP basin hosts a dense microbial mat that borders the rock glacier (Figure 1B). This mat was first described as approximately 500–600 m^2^ and 2–5 mm thick (Siegel et al., 1979). Microscopic investigation of the mat revealed an abundance of cyanobacteria phenotypically similar to *Oscillatoria*, in addition to the green algae *Chlorella* and *Dunaliella*, diatoms, invertebrates, and bacteria (Siegel et al., 1983). The extent to which the saturated brine of DJP might influence the mat is currently unknown, though it is possible that dried salts are deposited in the mat by aeolian processes (Diaz et al., 2018).

The genus *Massilia* was first described in 1998 in a report on the isolation of *Massilia timonae* from the blood sample of an immunocompromised patient (La Scola et al., 1998). The genus belongs to the Oxalobateraceae family and to date includes 61 validly published species (January 2023).^1^ *Massilia* species have been isolated from disparate environments across the globe and have been implicated in human disease (La Scola et al., 1998; Van Craenenbroeck et al., 2011; Kämpfer et al., 2012; Chiquet et al., 2015) and as a common member of rhizosphere communities of *Arabidopsis* (Tkacz et al., 2015) and wheat (Schlatter et al., 2020). Rhizosphere *Massilia* are of interest due to their possible role in plant development through the production of phytohormones and solubilization of phosphates, among other activities (Ofek et al., 2012; Schlatter et al., 2020). Several *Massilia* have also been isolated from the cryosphere, including from Arctic (Son et al., 2021; Dahal et al., 2021a), Antarctic (Holochová et al., 2020; Sedláček et al., 2022), and alpine regions (Shen et al., 2013, 2015; Guo et al., 2016; Gu et al., 2017; Wang et al., 2018).

While their ecological role in polar environments is not as well understood, *Massilia* species have been detected in diverse Antarctic niches. For example, *Massilia* ranged from 54 to 79% of culturable community members in both closed and open cryoconite holes in the Larsemann Hills, Dronning Maud Land, and Amery Ice Shelf regions (Sanyal et al., 2018). Sommers et al. (2019) identified *Massilia* as one of the ten most abundant sequences in cryoconite water collected from Taylor Glacier, a glacier within the MDV. These authors suggested that *Massilia* may play a role in cryoconite colonization. *Massilia* have also been isolated from microbial mats in both peninsular and continental Antarctica, including other ice-free regions such as the Schirmacher and Syowa Oasis (Peeters et al., 2012).

Here we characterize a red-pigmented *Massilia* species, strain DJPM01, isolated from the DJP microbial mat. We identified genomic evidence for environmental adaptations in strain DJPM01 and compared these findings with other Antarctic *Massilia* species as well as the taxonomic distribution of secondary metabolite biosynthetic gene clusters (BGCs) within the *Massilia* genus. We then analyzed cultures of strain DJPM01 for the presence and relative abundance of pigments when grown under ecologically relevant UV-A radiation exposure to further our understanding of microbial responses in extreme environments.

## 2. Materials and methods

### 2.1. Site description and sample collection

Samples of a microbial mat were collected in the South Fork of the Wright Valley at the terminus of a rock glacier in the Don Juan Pond (DJP) basin, Victoria Land, Antarctica (77.56293 S, 161.1751 E; Figure 1). Mat material was collected on 5 December 2018 using individually wrapped sterile scoops (SP Bel-Art; Wayne, NJ) and transferred into sterile Whirl-Pak bags (Whirl-Pak; Madison, WI). *In situ* temperature, pH, and salinity were measured at the time of collection using a vented Aqua TROLL 500 Multiparameter Sonde (*in-situ*), configured with pH/ORP, conductivity, rugged dissolved oxygen (RDO), and turbidity modules. Samples were stored and shipped cool (4°C) until later processing at the University of Tennessee (Knoxville, TN, United States).

### 2.2. Isolation and incubation conditions

Mat material was incubated in R2A broth (Difco; Becton, Dickinson and Company; Franklin, NJ) at ~10°C for 10 days, after which the broth was used to inoculate R2A agar plates (BD Difco; Franklin, NJ). Bright red-pigmented colonies appeared on the plate after 4 days of incubation at 10°C and were selected for further isolation through multiple restreaks on R2A agar. Gram staining and microscopy was performed on strain DJPM01 using the American Society of Microbiology Gram stain protocol (Smith and Hussey, 2005) and observed with an Axio Imager M2 microscope (Zeiss; Oberkochen, Germany). Motility assays were conducted by spot inoculation of strain DJPM01 onto 0.5% agar R2A plates. To assess resistance to UV-C light, R2A agar plates of strain DJPM01 were prepared and exposed UV light (254 nm) as described by Hirsch et al. (2004). Plates were exposed for 10, 20, 30, 60, 120, and 300 s and left to incubate in the dark at 10°C for 10 days. Strain DJPM01 was incubated in R2A broth under a nitrogen headspace to test for anaerobic growth. To test the effects of temperature on the growth rate of strain DJPM01, incubations were performed in 96-well plates. Briefly, 180 μL R2A broth was aliquoted into each well. Strain DJPM01 was grown to late log phase and inoculated into 48 wells of a 96-plate (10% inoculum). Plates were incubated at 4, 10, 15, 20, and 25°C, and full spectra were collected for each plate once per day for 14 days. Outer wells were removed from analysis to control for unequal levels of evaporation.

### 2.3. Phylogenetic analysis and whole genome comparison

For taxonomic identification, DNA was extracted from bacterial colonies using a DNeasy PowerSoil PowerLyzer Kit (Qiagen; Hilden, Germany) following the manufacturer’s protocol with the addition of a modified bead beating step as follows: 45 m sec^−1^ for 25 sec followed by 5 min rest at 10°C (FastPrep-24 Bead Lysis System; MP Biomedical; Santa Anna, CA). The 16S ribosomal RNA (rRNA) gene was amplified from extracted DNA using the following combination of primers: bacterial 27F (5’-AGAGTTTGATCCTGGCTCAG-3′; Weisburg et al., 1991) and 806R primers (5’-GGACTACNVGGGTWTCTAAT-3′; Apprill et al., 2015) and 515F (5’-GTGCCAGCMGCCGCGGTA-3′; Caporaso et al., 2011) and 1492R primers (5’-GGTTACCTTG TTACGACTT-3′; Weisburg et al., 1991). The resulting PCR products were purified using a Wizard SV Gel and PCR Clean-Up System Kit (Promega; Madison, WI) before Sanger sequencing, performed by Eurofins Genomics (Louisville, KY). Returned sequences were aligned using Geneious v6.1.8 (for a sequence length of 1,388 bp; Kearse et al., 2012) and compared to the NCBI nucleotide collection type material database (Federhen, 2012). Publicly available sequences for *Massilia* 16S rRNA genes were obtained from the RefSeq database (O'Leary et al., 2016), aligned, trimmed to the length of the shortest sequence (1,335 bp), and used to construct a maximum likelihood tree in MEGA v11.0.13 (Tamura et al., 2021). Additionally, strain DJPM01 was compared to all NCBI reference genomes within the Oxalobateraceae family using whole genome average nucleotide identity (ANI) through the enveomics collection (Rodriguez-R and Konstantinidis, 2016). Digital DNA–DNA hybridization (DDH) for all reference genomes available in the Oxalobacteraceae family was calculated using the Genome-to-Genome Distance Calculator (GGDC) v3.0 (Meier-Kolthoff et al., 2022). A final phylogenetic tree of RefSeq reference genomes was generated using OrthoFinder v2.5.4 (Emms and Kelly, 2019) with default settings. Estimation of abundance was performed using a 16S community amplicon sequence variant (ASV) library generated from the DJP mat (Schuler, 2022) using the Basic Local Alignment Search Toolkit (BLAST; Altschul et al., 1990). The final 16S rRNA gene sequence for strain DJPM01 was uploaded to GenBank under accession number OQ346149.

### 2.4. Genome sequencing, assembly, and annotation

Total genomic DNA was extracted from microbial biomass using a cetrimonium bromide (CTAB) buffer/organic phase extraction protocol (as described by Campen, 2015) for whole genome sequencing. Quality and quantity of extracted DNA was determined by a Qubit 4 fluorometer (Invitrogen Carlsbad, CA) and NanoDrop 2000 spectrophotometer (Thermo Scientific; Waltham, MA, United States). High molecular weight genomic DNA (25 ng/μL) was sequenced by MR DNA (Shallowater, TX). Library preparation was performed using the Nextera DNA Flex Library Preparation Kit (Illumina Inc.; San Diego, CA) and quality was confirmed using the Agilent 2,100 Bioanalyzer (Agilent Technologies; Santa Clara, CA). The library was diluted to 0.6 nM before sequencing on the NovoSeq platform (Illumina; San Diego, CA; paired end, 500 cycles). Sequencing adapters and low-quality reads were trimmed from using Trimmomatic v0.36 (Bolger et al., 2014) on default parameters. Trimmed reads were assembled using Unicycler v0.4.8 (Wick et al., 2017) with a maximum contig size of 500 bp and assembly quality was assessed using QUAST v5.0.2 (Gurevich et al., 2013) and checkM v1.1.3 (Parks et al., 2015). The genome draft was annotated by Prokka v1.13.7 (Seemann, 2014) and the resulting annotation was analyzed using GhostKOALA v2.2 (Kanehisa et al., 2016). Putative CRISPR-Cas sequences were identified using CRISPRCasFinder v4.2.20 (Couvin et al., 2018) and putative prophage sequences were identified by VirSorter2 v2.2.3 (Guo et al., 2021), both using the Proksee interface.^2^ Putative secondary metabolite BGCs were identified *via* antiSMASH v6.0.0 (Blin et al., 2021) using default parameters. RefSeq *Massilia* genomes were annotated using Prokka v1.13.7, GhostKOALA v2.2, and antiSMASH v6.0.0 and all *Massilia* genomes in both NCBI and JGI databases were aligned against the *pig* operon of *Serratia* sp. strain ATCC 39006 to search for prodigiosin-specific biosynthetic genes (Harris et al., 2004). Putative prodigiosin operons were compared by pairwise alignment using SimpleSynteny v1.5 (Veltri et al., 2016). Statistical analyses of gene abundance were performed using R v4.1.2 (R Core Team, 2018). The final draft genome assembly was uploaded to GenBank under accession number JAQOZV000000000.

### 2.5. Biomass irradiation and extraction of pigments produced under UV light

A lawn of strain DJPM01 was grown on R2A agar plates in quadruplicate and incubated under a UV-A light (320–400 nm, peak absorbance 395 nm; Supplementary Figure S1; SANSI Lighting; Shanghai, China). Nalgene petri dishes (Thermo Scientific; Waltham, MA) made of polymethylpentene were utilized to increase UV penetration compared to standard polystyrene petri dishes (Peng et al., 2011). Control plates were inoculated, wrapped in foil, and placed inside two black bags prior to incubation. Uninoculated media controls were also incubated under both light and dark treatments. All plates were incubated at 10°C for 11 days. Following incubation, agar was sterilely sliced into strips for storage at −80°C. Agar strips from each plate were extracted four times with 10 mL of high performance liquid chromatography (HPLC)-grade ethyl acetate (Fisher Scientific; Hampton, NH) and sonicated for 10 min. Organic phases were pooled and diluted to 50 mL with ethyl acetate. Authentic prodigiosin (1) standard (P0103; Sigma-Aldrich, St. Louis, MO) was diluted 100 μg mL^−1^ in liquid chromatography/mass spectrometry (LC/MS)-grade methanol (Fisher Scientific; Hampton, NH) and exposed to UV-A radiation overnight to investigate potential breakdown products. UV-A exposed and unexposed prodigiosin (1) standard solutions were diluted to 10 μg mL^−1^ prior to LC/MS analysis.

### 2.6. LC/MS analyses

Extracts were analyzed on a Thermo Scientific (Waltham, MA) Q-exactive HF-X Hybrid Quadrupole-Orbitrap mass spectrometer interfaced with a Vanquish Horizon Ultra high performance liquid chromatography (UHPLC) system and VH-D10-A UV detector using a Waters (Milford, MA) HSS T3 C18 column (1.8 μm, 2.1 × 100 mm) with a VanGuard FIT precolumn. Using a flow rate of 0.5 mL min^−1^, samples (2 μL) were injected onto the column prewarmed to 40°C. UHPLC separation was achieved using the following parameters: 2% acetonitrile: 98% water with 0.1% formic acid for 1 min, 2–40% over 4 min, 40–98% over 3 min, 98–2% over 0.2 min, and 2% for 2 min. The UV–Vis data were collected at 534 nm. The following electrospray ionization (ESI) settings were used in probe position D: 40 sheath gas flow, 8 auxiliary gas flow, 1 sweep gas flow, 3.5 kV spray voltage, 380°C capillary temperature, 50 radiofrequency (RF) funnel level, and 350°C auxiliary gas temperature. Data were evaluated using MS-DIAL (Tsugawa et al., 2015) and MPACT (Samples et al., 2022). See supplementary information for data processing methods.

A Waters Synapt G2-Si Q-ToF mass spectrometer coupled to an Acquity H-Class ultra performance liquid chromatography (UPLC) system was employed for LC/MS analysis of select samples and prodigiosin (1) standard using the previously described column and gradient. Positive mode MS^E^ acquisition was used, using 0.1 s MS1 survey scans from *m/z* 150–2000 in resolution mode, followed by 0.1 s high energy MS2 scan from *m/z* 50–2000. Data were acquired using the following parameters: 2 kV capillary voltage, 100°C source temperature, 20 V sampling cone, 800 L h^−1^ desolvation gas flow, 80 V source offset, and 30 V MS2 collision energy. Real-time mass correction used a lockspray of 400 pg μL^−1^ leucine enkephalin solution (1:1 methanol to water solution with 0.1% formic acid). A 10 μL injection volume was used in the closed loop configuration.

## 3. Results

### 3.1. Sample collection and isolation

At time of collection, the water within the mat was 7.7°C; collection site pH was 7.2 and salinity was 0.4 parts per thousand (ppt). Following isolation, the purity of the resulting axenic culture was confirmed by 16S rRNA gene analyses and microscopy, which revealed a single rod-shaped morphology. The resulting red-pigmented isolate was designated strain DJPM01.

### 3.2. Characterization and growth

**Figure 2:**
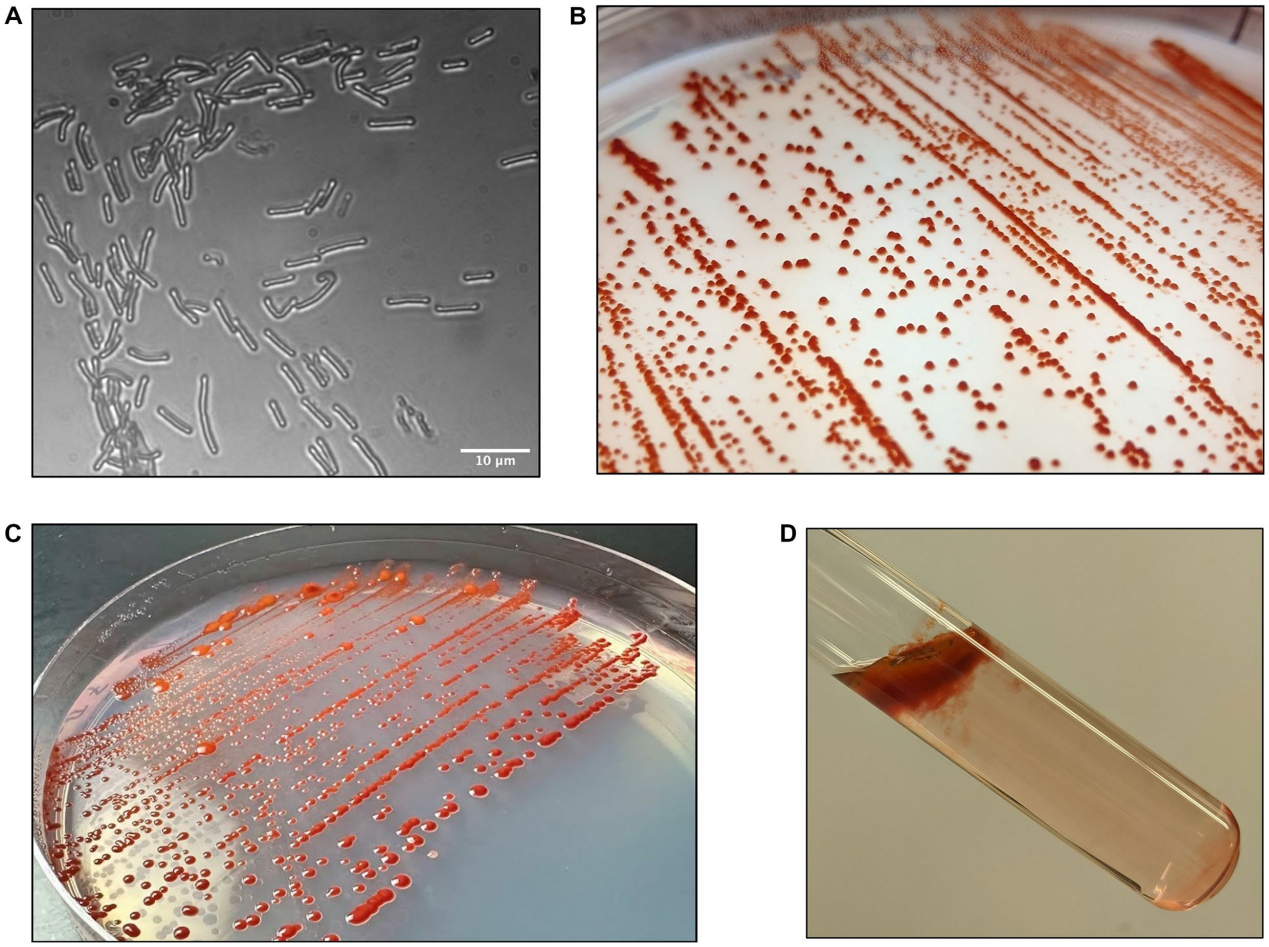
Morphology of strain DJPM01. **(A)** Light microscopy image of strain DJPM01 using phase shift. Cells were rods and 4.65 μm in length on average. **(B,C)** Strain DJPM01 grown on R2A agar at 10 **(B)** and 15 **(C)** °C. Colonies were robust and resisted removal from the agar. Bright red pigmentation was present at all temperatures. **(D)** Pellicle formation by strain DJPM01 in R2A broth, held at an angle.

Growth of strain DJPM01 on R2A yielded tall, convex, scarlet colonies with entire margins that spread outwards over time (Figure 2). Colonies were resistant to removal from the surface of the agar and remained aggregated when transferred to broth culture, unless thoroughly agitated through vortexing. Strain DJPM01 was determined to be Gram-negative using the standard Gram stain protocol (Smith and Hussey, 2005). Cells were rod-shaped and 4.65 μm in length on average (Figure 2A). Cultures exhibited growth from 4 to 25°C, but not at 30°C (Supplemental Figure S2). The fastest growth occurred at 10°C with a rate (μ) of 0.25 ± 0.04 day^−1^. Colonies were pigmented under all culture conditions, with some variation in color intensity and hue at warmer temperatures (Supplemental Figure S2). Growth was not detected under anaerobic conditions. Strain DJPM01 grew on R2A agar adjusted to pH 10.5. Abundance of colony forming units decreased following 30 s of exposure to UV-C radiation, however colonies were present on plates irradiated for up to 120 s. Evidence for motility was observed when grown on low density R2A agar. When grown in R2A broth, strain DJPM01 formed a robust pellicle at the surface and produced extracellular polymeric substances (Figure 2D).

### 3.3. Phylogenetic analysis

Analysis of the trimmed 16S rRNA gene sequence (1,335 bp) placed strain DJPM01 within the *Massilia* genus (Supplementary Figure S3). Strain DJPM01 shared highest sequence identity with *M. violaceinigra* strain B2 (CP024608, 99.87%) originally isolated from glacial permafrost in the Xinjiang region of China (Wang et al., 2018). Strain DJPM01 also shared high sequence identity with several other cryosphere associated *Massilia* species, including *M. aquatica* strain CCM 8693 (MN612031, 99.87%), *M. rubra* strain CCM 8692 (MN611986, 99.80%), *M. frigida* strain CCM 8695 (MN612047, 99.80%), *M. mucilaginosa* strain 8,733 (MN612043, 99.67%), and *M. atriviolacea* strain SOD (NR_171529, 99.40%). Of these six isolates, four were isolated from James Ross Island on the Antarctic Peninsula (*M. frigida, M. aquatica, M. rubra,* and *M. mucilaginosa*; Holochová et al., 2020). *M. atriviolacea,* was first isolated from agricultural soils from Hefei, China (Yang et al., 2019). Comparison of the 16S rRNA sequence to the available DJP basin mat 16S ASV library suggests that strain DJPM01 is present in low abundance with 2,110 reads or ~0.03% relative abundance (Schuler, 2022).

**Figure 3:**
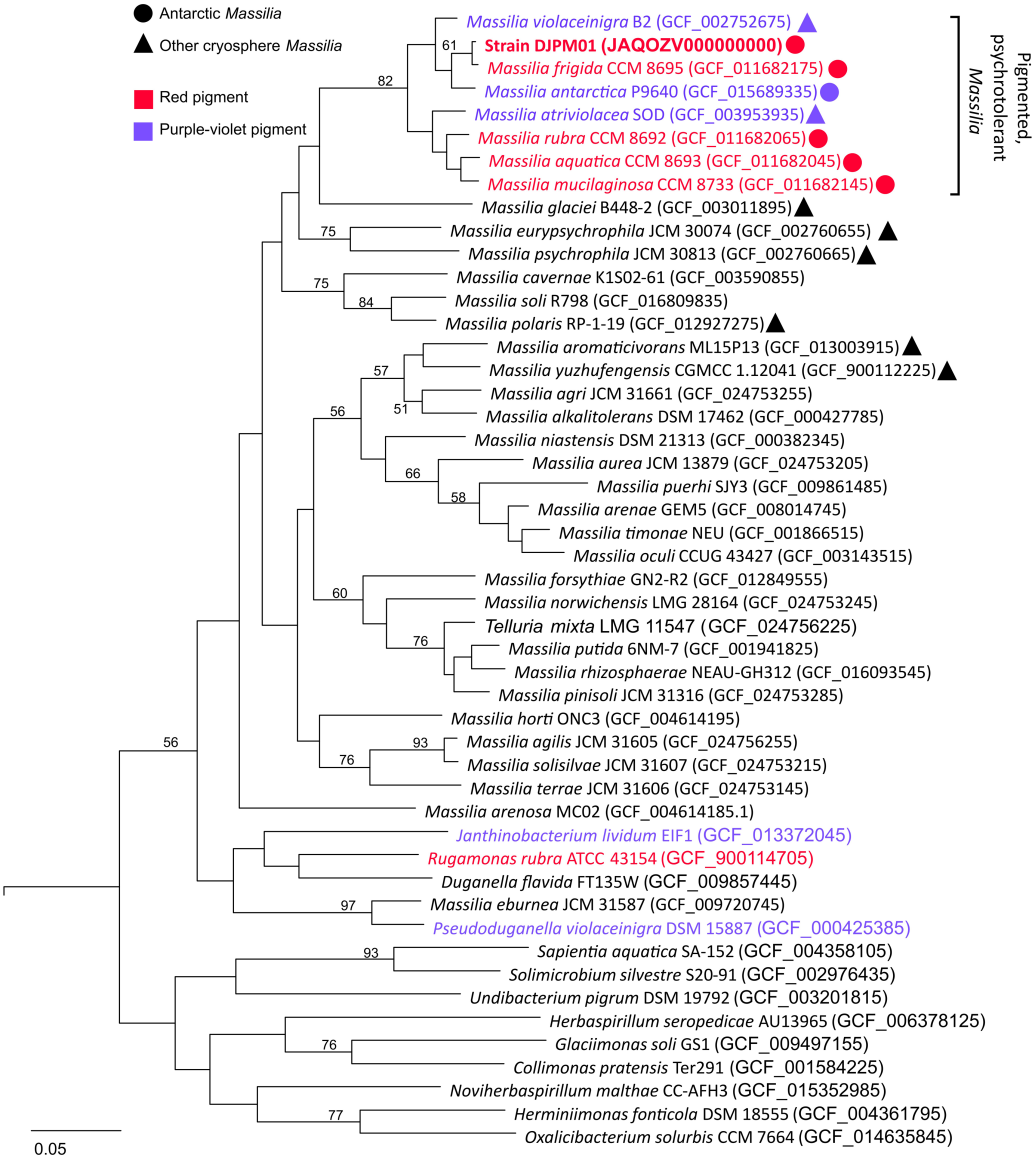
Multiple-gene tree of Oxalobacteraceae family members. Tree constructed using OrthoFinder v2.5.4 (Emms and Kelly, 2019). All *Massilia* with phenotypic and genotypic evidence for red (prodigiosin) or purple-violet (violacein) pigment production form a clade with >87% ANI between any given pair. All members of this clade were isolated cryosphere environments, including all five Antarctic *Massilia*. All *Massilia* genomes cluster into a single clade, with the exception of *M. eburnea*, originally identified as a species of *Pseudoduganella* (Lu et al., 2020). Node values represent bipartition support values >50. An Antarctic *Shewanella* species (strain BF02_Schw) was used as an outgroup (DQ677870.1).

Whole genome comparison placed strain DJPM01 within a distinct clade (≥87% ANI between clade members) including all currently identified *Massillia* with either a red or purple pigmented phenotype (Figure 3; Supplementary Table S1). This clade includes the six *Massilia* strains with highest 16S rRNA gene similarity to strain DJPM01, as well as *M. antarctica* strain P9640 (OM243919; Sedláček et al., 2022), which was also isolated from James Ross Island. All members of this clade have been shown to grow at or below 4°C, regardless of isolation locale. Strain DJPM01 shared highest ANI with *M. frigida* strain CCM8695 (97.36% identity). High sequence identity of strain DJPM01 to *M. antarctica* strain P9640 (91.92%) and *M. violaceinigra* strain B2 (91.38%) were also noted. Additionally, *M. mucilaginosa* strain CCM8733, *M. atriviolacea* strain SOD, *M. rubra* CCM 8692 (GCF 011682065), and *M. aquatica* CCM 8693 had relatively high sequence identity (87.94–88.41% similarity to strain DJPM01). Digital DNA–DNA hybridization (DDH) showed 75.6% similarity between strain DJPM01 and *M. frigida* strain CCM8695 (Supplementary Table S1).

### 3.4. Genomic analyses

**Figure 4:**
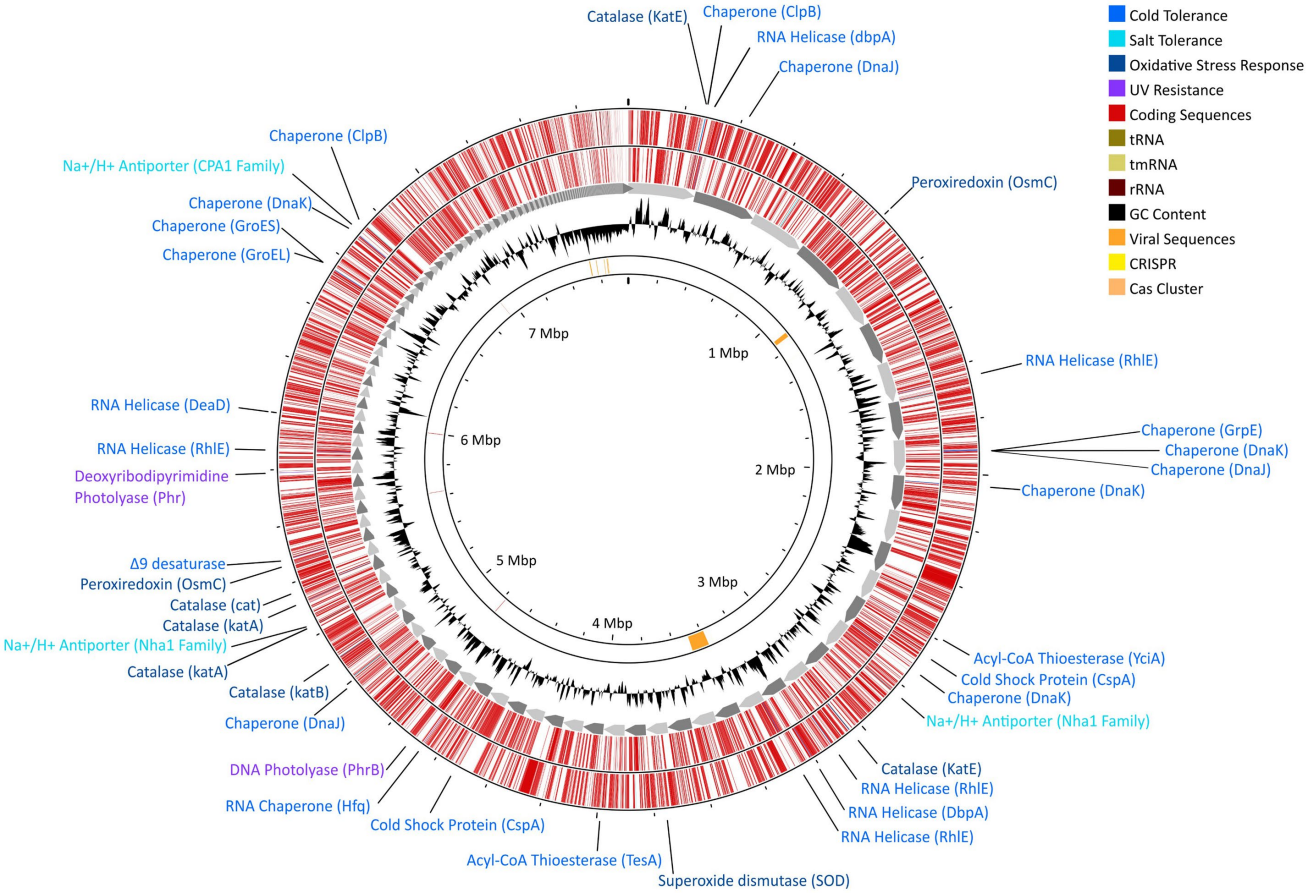
Gene map for strain DJPM01 highlighting proposed environmentally-relevant genes. Contigs are sorted by length and represented by grey arrows. Outer tracks depict coding sequences as annotated by Prokka v1.13.7 (Seemann, 2014), separated by strand. Genes related to environmentally-relevant stress adaptation and response are highlighted and labeled. Results of VirSorter2 v2.2.3 (Guo et al., 2021) and CRISPRCasFinder v4.2.20 (Couvin et al., 2018) are present on the inner ring.

Whole-genome sequencing resulted in 8,995,221 paired reads of which 8,693,443 (96.6%) pairs survived trimming with 0 N’s. Assembly yielded a genome 7.7 Gb in size across 236 contigs with 99.80% completeness (573 of 574 marker genes present) and 1.64% contamination (10 marker genes with two copies each; Figure 4). Following annotation, 6,626 predicted protein coding genes were identified, 3,037 of which had a non-hypothetical function (45.8% functional annotation). Of these genes, the largest functional categories within the KEGG database were protein families: signaling and cellular processes (361 genes), environmental information processing (339 genes), genetic information processing (339 genes), and carbohydrate metabolism (248 genes). The genome contains complete pathways for glycolysis and the TCA cycle, oxidative phosphorylation, flagellar motility, and denitrification. Additionally, we detected 9 putative CRISPR arrays and 4 Cas clusters (Figure 4). Both putative single- and double-stranded DNA phage sequences were found throughout the genome.

Strain DJPM01 has genomic evidence for adaptation to cold environments (Figure 4; Supplementary Table S2). The genome contains genes homologous to those encoding protein chaperones, including two copies of ClpB (Mogk et al., 2003) and DnaJ (Qiu et al., 2006), three copies of DnaK (Thompson et al., 2012), and one copy each of GroEL (Lenz and Ron, 2014), GroES (Takei et al., 2012), and GrpE (Wu et al., 1996). There was also evidence for genes encoding cold shock proteins involved in repairing misfolded mRNA, such as ATP-dependent RNA helicases DpbA (Tsu et al., 2001), DeaD (Prud’homme-Généreux et al., 2004), and three copies of RhlE (Bizebard et al., 2004). Strain DJPM01 contained two copies of genes putatively encoding the cold shock protein CspA (La Teana et al., 1991). Three genes were also associated with the formation of unsaturated fatty acids, including a Δ9-desaturase (Li et al., 2009) and two acyl-CoA thioesterases, TesA (Cho and Cronan, 1993) and YciA (Postle and Good, 1985).

Several genes putatively involved in osmotic and oxidative stress were present as well (Figure 4; Supplementary Table S2). Strain DJPM01 has genomic evidence of several cation/proton antiporters in the NhaA (Vimont and Berche, 2000) and CPA1 families (Utsugi et al., 1998). Both the biosynthetic pathway and transporters for the compatible solute glutamate are present (Saum et al., 2006). Strain DJPM01 also contained genes for superoxide dismutase (Fee, 1991) and peroxiredoxin (Weber et al., 2006), which are enzymes known to combat oxidative stress, in addition to six copies of the catalase gene (von Ossowski et al., 1991). Several genes were associated with DNA repair (Figure 4; Supplementary Table S2). Strain DJPM01 encodes for several copies of genes for nucleotide excision, mismatch repair, and homologous and non-homologous recombination. The genome also encodes for two proteins associated with light-dependent DNA repair, deoxyribodipyrimidine photolyase (Sancar et al., 1984) and DNA photolyase (Zhang et al., 2013).

Annotation by antiSMASH (Blin et al., 2021) revealed a total of 17 putative biosynthetic gene clusters (BGCs), including high similarity matches to characterized gene clusters for aryl polyene APE_Ec_ (36%), violacein (100%), cephamycin C (36%), myxochelin (41%), iso-migrastatin (45%), and prodigiosin (42%; Supplementary Table S3). Of these genes, aryl polyenes have been proposed to mediate oxidative stress (Cimermancic et al., 2014), myxochelins are iron-chelating siderophores (Li et al., 2008), cephamycin C is a β-lactam antimicrobial (Alexander and Jensen, 1998), and iso-migrastatin is a glutaride-containing cytotoxic agent (Ju et al., 2005). The pigments violacein (blue-violet) and prodigiosin (1; red) have both been described to have antibiotic properties and UV-protective effects (Danevčič et al., 2016; Cauz et al., 2019). Closer analysis of the iso-migrastatin BGC using PRISM v4.4.5 (Skinnider et al., 2020) revealed this cluster encodes genes which are 58–72% identical (based on amino acid sequence) to those involved in the biosynthesis of the antifungal gladiofungin, however the cluster was on the end of a contig, missing roughly 15% of the third polyketide synthase gene (Supplemental Figure S4; Niehs et al., 2020).

**Figure 5:**
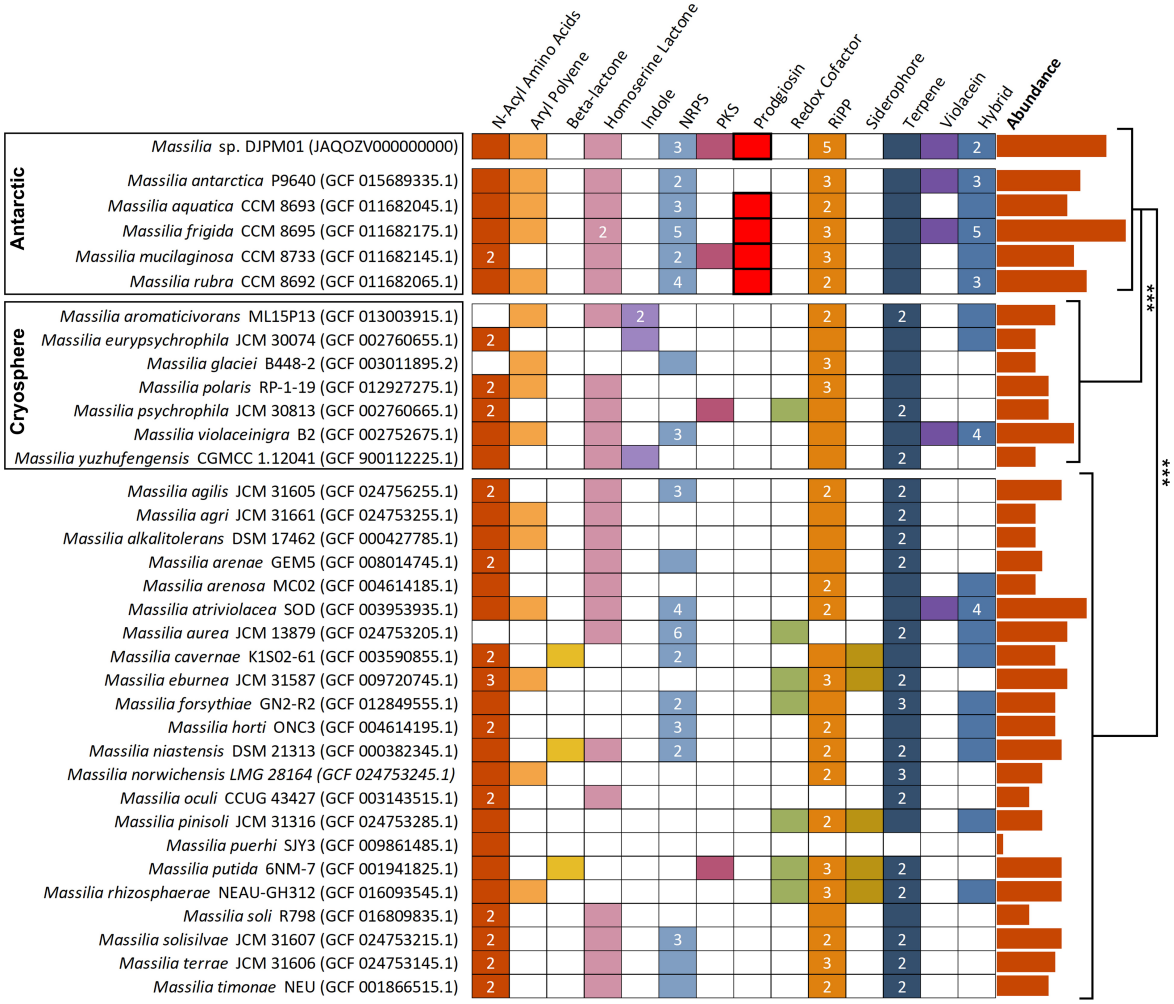
Annotated secondary metabolite BGS in *Massilia* reference genomes, sorted by isolation environment. Clusters have been sorted into broad categories, with hybrid clusters containing motifs of multiple categories (i.e., PKS-NRPS). Numbers represent the total abundance of clusters when multiple are present within the genome. Prodigiosin and violacein BGCs were confirmed *via* BLAST. Absolute abundance of clusters are plotted to the right of the figure and are significantly different between isolation environments (Tukey). NRPS, nonribosomal peptide synthetase; PKS, polyketide synthase; RiPP, ribosomally synthesized and post-translationally modified peptide.

To understand how the diversity of BGCs in DJPM01 compares to other *Massilia* genomes, putative secondary metabolite BGCs were identified in representative *Massilia* genomes from RefSeq using antiSMASH (Figure 5; O'Leary et al., 2016; Blin et al., 2021). Antarctic *Massilia* genomes contained an average of 14.5 secondary metabolite BGCs per genome, significantly higher than both mesophilic *Massilia* (9.2 BGCs per genome; Tukey, *t* = −4.95, *p* < 0.001) and Arctic and alpine *Massilia* (7.8 BGCs per genome; Tukey, *t* = −4.31, *p* < 0.001). This trend was still significant when normalized to genome size (ANOVA, *F* = 4.27, *p* = 0.023) and number of coding sequences per genome (ANOVA, *F* = 4.38, *p* = 0.021; Supplementary Table S4). Strain DJPM01 was the only *Massilia* to contain the putative gladiofungin polyketide gene cluster. The BGC for prodigiosin was only identified in Antarctic *Massilia* species (*M. aquatica* CCM 8693, *M. frigida* CCM 8695, *M. mucilaginosa* CCM 8733, and *M. rubra* CCM 8692).

Genes for *Serratia* sp. strain ATCC 39006 prodigiosin BGC were aligned to all *Massilia* genomes available on NCBI (Harris et al., 2004). Only five genomes were identified to contain BGCs with high sequence identity to the prodigiosin BGC, with the highest bit scores for *pigC*, *pigD*, and *pigE* (Supplementary Figure S5A). Of these five organisms, four were *Massilia* species originally isolated from James Ross Island. The fifth genome was reported as *M. violaceinigra* strain sipir (GCF 022811105.1) in NCBI; the metadata associated with this entry indicated it was isolated from freshwater. Our analyses indicated this genome had >97% ANI to the Antarctic *M. aquatica* CCM 8693, which also has genotypic and phenotypic evidence for prodigiosin production. Given the uncertainty about the phylogeny of this strain we excluded it from further analyses. The four Antarctic *Massilia* prodigiosin BGCs had high sequence identity (93.59–100.00% identity) at the hypothetical gene level to strain DJPM01 (Supplementary Figure S5B).

Gene annotations were compared between strain DJPM01 and *M. frigida* stain CCM 8695. Both strains shared 1990 genes with non-hypothetical functions (97.9%), with 41 genes unique to strain DJPM01 and 51 genes unique to *M. frigida*. Genes unique to strain DJPM01 included two genes associated with lipopolysaccharide biosynthesis (*hddA* and *hddC*), one copy of fatty acid elongation gene *fabI*, and three genes associated with toxin-antitoxin systems (*fitA, fitB,* and *yefM*). Both genomes contain genes associated with type IV pilin production, multifunctional fibers produced on cell surfaces, though *M. frigida* does not have a copy of *mshC* (Toma et al., 2002). An additional 91 genes had a higher copy number in strain DJPM01 and 102 genes had lower copy number in strain DJPM01. Strain DJPM01 had 2 additional copies of genes for catalase, and four fewer copies of the fatty acid biosynthesis gene *fabB*. When compared to non-Antarctic species, Antarctic *Massilia* tended to, on average, have slightly more genes associated with fatty acid biosynthesis (especially *fab* genes; 4 more genes), protein chaperones (1.4 more genes), and catalase (1.4 more genes).

### 3.5. Confirmation of prodigiosin in strain DJPM01

**Figure 6:**
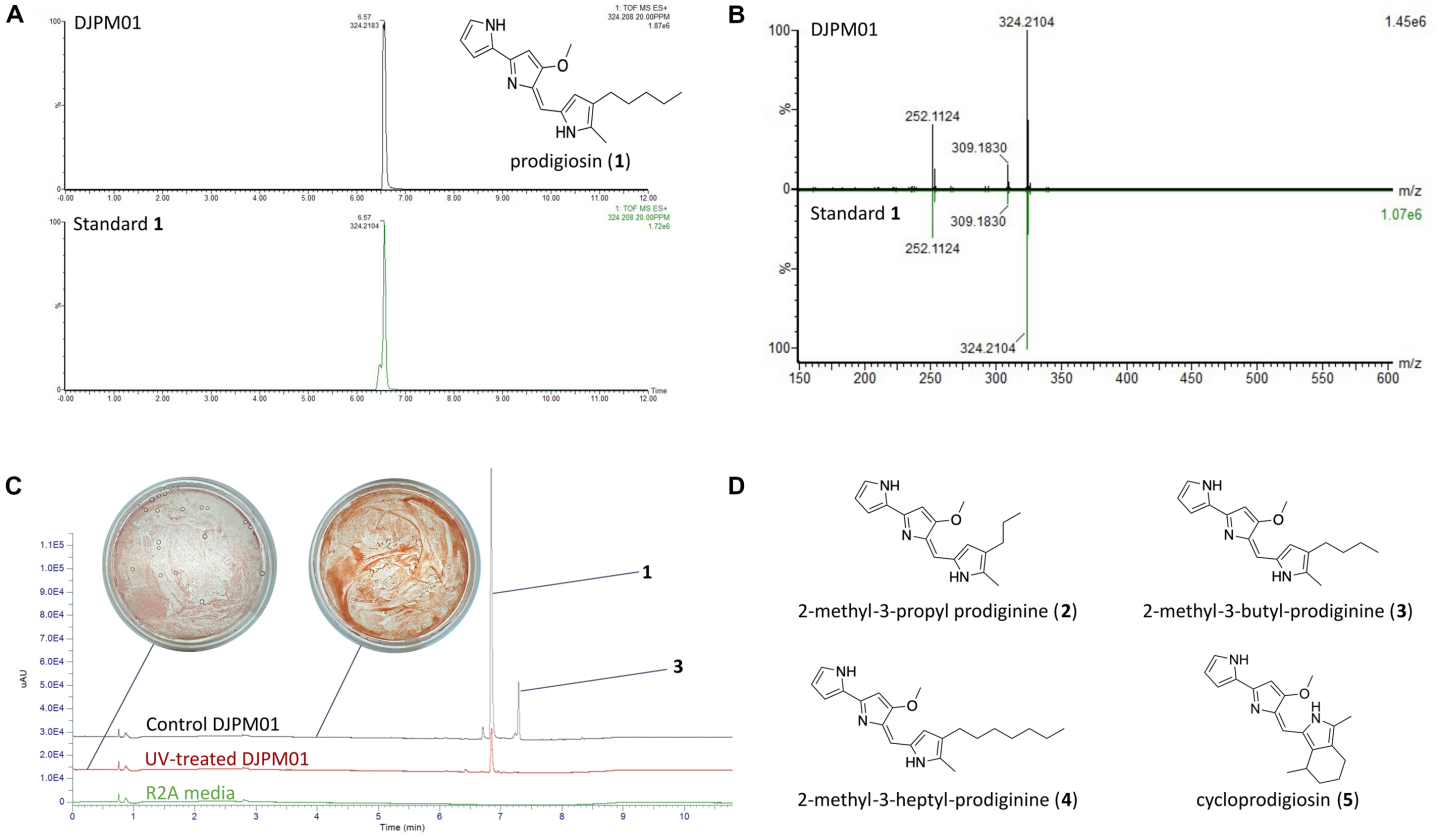
Detection of prodigiosin and derivatives in strain DJPM01 extracts. **(A)** Extracted ion chromatograms (m/z 324.208) of prodigiosin (1) in DJPM01 R2A agar extracts (black) compared to a prodigiosin standard (green). **(B)** Mirrored MS/MS mass spectra of prodigiosin (1) in DJPM01 agar extracts and prodigiosin standard. UV–Vis spectra of prodigiosin (1) in DJPM01 agar extracts (black) and prodigiosin standard (green) showing identical spectra. **(C)** Stacked UV trace diagram for plate extracts from control and UV-treated cultures. Dominant peaks corresponding to prodigiosin (1) and 2-methyl-3-butyl-prodiginine (3). Images of UV-exposed (left) and control (right) plates following 11 days of incubation. **(D)** Molecular structures of detected prodigiosin derivatives.

LC/MS and UV–Vis spectroscopic analyses of extracted DJPM01 agar plates resulted in the detection of prodigiosin (1), which was confirmed with an authentic standard (Figures 6A,B). Prodigiosin (1, *m/z* 324.2072; calculated for C_20_H_26_N_3_O [M + H]^+^, 324.2076) was detected with a characteristic UV–Vis spectrum (λ_max_ = 540 nm; Figures 6A,B; Supplementary Figure S6; Andreyeva and Ogorodnikova, 2015). MS/MS spectra of the peak identified as prodigiosin (1) were congruent with the fragmentation pattern of the authentic standard. Furthermore, a number of distinct peaks with similar UV–Vis absorbances were detected in chromatograms with masses consistent with prodiginines based on mass defect values and even [M + H]^+^ masses. Metabolomic analyses indicated the presence of numerous features consistent with those of 2-methyl-3-propyl prodiginine (2), 2-methyl-3-butyl prodiginine (3), 2-methyl-3-heptylprodiginine (4), cycloprodigiosin (5), and other uncharacterized prodiginines (Figure 6D; Supplementary Table S5).

### 3.6. Effects of UV light on the production of prodigiosin and other secondary metabolites

**Figure 7:**
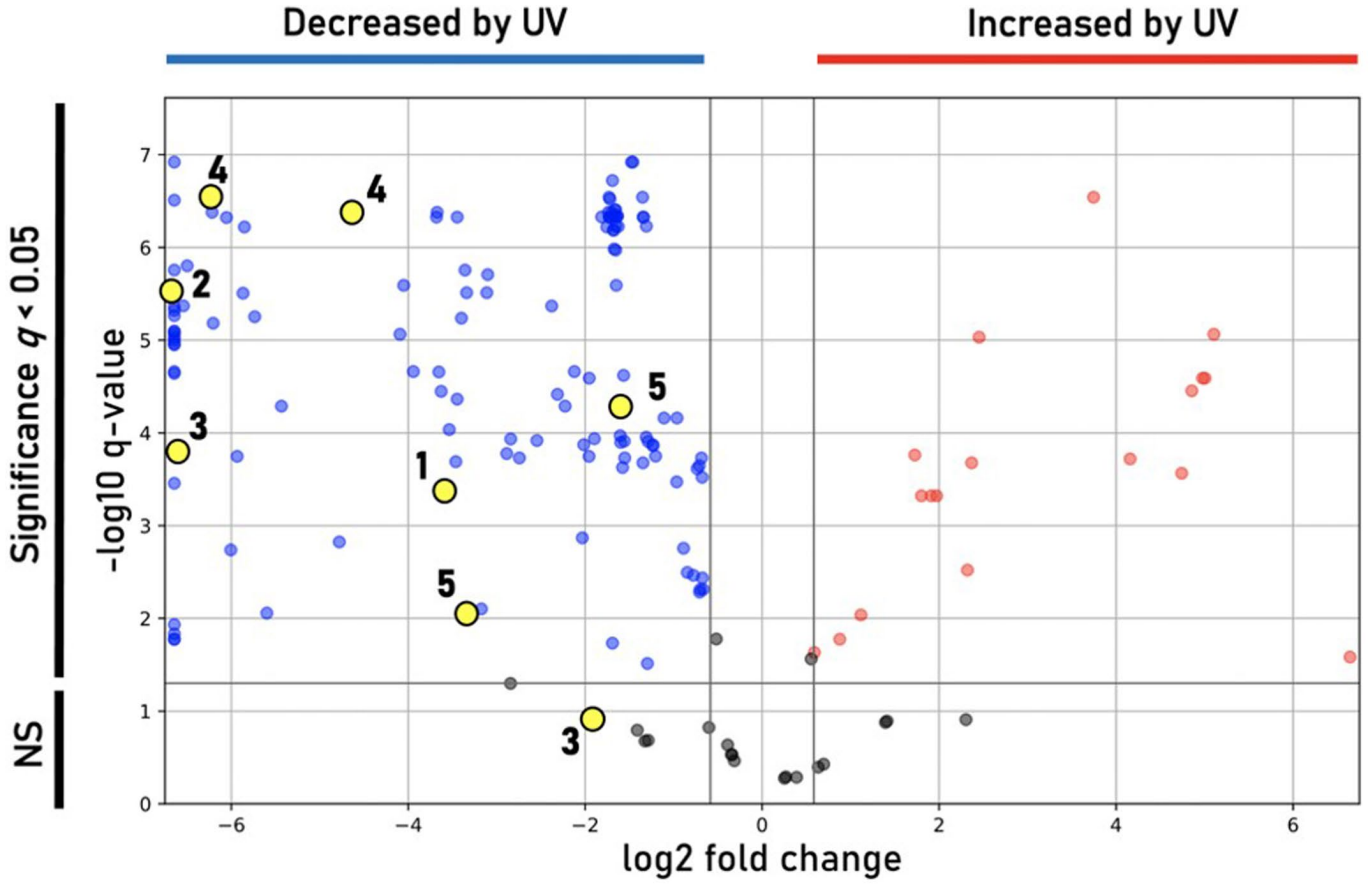
Differential features observed under UV treatment. Volcano plot showing statistically significant (*q* < 0.05) features in DJPM01 grown on R2A agar that are either increased (red) or decreased (blue) relative to control samples that were not irradiated with UV-A light. Numbered and unnumbered yellow dots correspond to known features and unknown, prodiginine-related features, respectively.

Incubation under UV light yielded a less-saturated, darker-hued pigmentation compared to control plates grown in the dark, with no UV exposure (Figure 6C). LC/MS analyses of UV-treated strain DJPM01 indicated statistically significant differences between UV-treated, control, and media blank samples (Supplementary Figure S7). A statistically significant decrease was observed in prodigiosin (1; *q* < 0.001) and other metabolites (Figures 6C, 7). Mass spectral analyses suggested that these uncharacterized prodiginines may be cyclized, reduced, oxygenated, and otherwise modified variations of prodigiosin. LC/MS analysis of a UV-treated prodigiosin (1) standard indicated a conversion of the compound to a non-pigmented degradation product with an *m/z* of 354.2169 [M + H]^+^ and probable molecular formula of C_21_H_28_N_3_O_2_ (Supplementary Figure S8). Additionally, several other features with mass shifts consistent with addition of CH_2_O to major prodiginines were observed. The only compound unique to the UV-treated strain DJPM01 was glycerophosphoserine (*m/z* 522.2831; calculated [M + H]^+^ for C_24_H_45_NO_9_P, 522.2832;). Masses congruent to those of arylpolyenes and migrastatin (*m/z* 512.2617; calculated [M + H]^+^ for C_24_H_45_NO_9_P, 522.2832) were also detected. No LC/MS or UV–Vis spectroscopic evidence of other pigments was found.

## 4. Discussion

*Massilia* species have been identified in diverse ecological niches across Antarctica, though little is known about their function within cold systems. We isolated strain DJPM01 from a microbial mat which sits adjacent to one of the saltiest aquatic features on Earth, the hypersaline Don Juan Pond in the McMurdo Dry Valleys (Toner et al., 2017). We identified this aerobic, Gram negative bacterium as a member of the *M. frigida* species. This strain was isolated on R2A agar, which is commonly utilized to cultivate bacteria from Antarctic environments, though growth on this medium is not an indicator of abundance or importance to a given system (Pulschen et al., 2017). Strain DJPM01 is estimated to be present at low abundance (~0.03%) in the established microbial mat community; however, abundance and ecological importance are not mutually exclusive, as highlighted with the rare biosphere concept (Lynch and Neufeld, 2015). Members of the *Massilia* genus have been of interest for their agricultural and pharmaceutical applications (Lewin et al., 2021; Dahal et al., 2021b). Thus, we examined both potential adaptations to ecologically relevant conditions and production of microbial secondary metabolites, including the pigment prodigiosin, in strain DJPM01.

### 4.1. Phylogeny and ecophysiology of an Antarctic *Massilia* strain

Modern prokaryotic species concepts strive to incorporate both the advanced sequencing tools available to interrogate microbial genomes as well as phenotypic assays (Rosselló-Móra and Amann, 2015). While specifics may be debated, it is generally agreed that species exhibit a large degree of genomic and phenotypic coherence (Rosselló-Móra and Amann, 2015). Conservative estimates indicate that a DDH of >70% (Tindall et al., 2010), ANI threshold of 95–96% (e.g., Konstantinidis and Tiedje, 2005; Konstantinidis et al., 2006), and 16S rRNA gene sequence identity of >98% (Kim et al., 2014) is appropriate for delineating prokaryotic species for taxonomic purposes. While strain DJPM01 has >99% sequence identity with several *Massilia* type strains, highest ANI (97.36%) and DDH (75.6%) were found with *M. frigida* strain CCM8695. Thus, we conclude that strain DJPM01 is a strain of this species group.

Strain DJPM01 shares phenotypic similarities with *M. frigida* strain CCM 8695 and other psychrotolerant strains, including colony morphology, motility, inability to grow at temperatures at or above 30°C, and sensitivity to UV-C irradiation (Holochová et al., 2020). Strain DJPM01 was more tolerant of alkaline growth conditions compared to reported observations of both psychrotolerant and mesophilic *Massilia*, though several *Massilia* species are also able to grow on media above pH 10, including *M. alkalitolerans*, which can survive on media adjusted to pH 12 (Xu et al., 2005). Based on 16S rRNA gene and whole-genome comparisons, strain DJPM01 forms a clade with seven named psychrotolerant *Massilia*, including the five described Antarctic species (Wang et al., 2018; Yang et al., 2019; Holochová et al., 2020; Sedláček et al., 2022). Of the Antarctic *Massilia* with published genome sequences, four have been described as producing a pink-red pigment when grown on R2A agar (Holochová et al., 2020) and contain the complete pathway for prodigiosin biosynthesis. The fifth, *M. antarctica* strain P9640, produces a blue-purple pigment, likely violacein (Sedláček et al., 2022). However, *Massilia* isolates beyond this clade (<82% ANI) have only been described as either unpigmented or as producing a yellow pigment, which could be carotenoids based on the putative BGCs present in their genomes (Gallego et al., 2006; Zhang et al., 2006).

### 4.2. Genomic adaptations to life in an Antarctic microbial mat

The genome of strain DJPM01 contains several adaptations to life in an Antarctic microbial mat. Low temperatures can negatively impact transcription and translation, both through decreases in reaction rate and through changes in the secondary structure of DNA, RNA, and proteins (De Maayer et al., 2014). Strain DJPM01 encodes multiple sets of genes associated with either preventing the formation of transcription-inhibiting RNA secondary structures (cold shock proteins) or unraveling the structures that do form (RNA helicases; Siddiqui et al., 2013). Similarly, strain DJPM01 has genes encoding for several protein chaperones to ensure correct folding of polypeptide chains. Other genes, such as *fab* genes and a putative Δ9-desaturase, are involved in the biosynthesis of unsaturated fatty acids to increase membrane fluidity at low temperature (De Maayer et al., 2014). Cold-adapted organisms synthesize compatible solutes to prevent the formation of ice crystals within the cell and combat changes in salinity associated with freezing temperatures (Chin et al., 2010). Genes involved in the biosynthesis and transport of the compatible solute glutamate are present within the genome, as well as several cation/proton antiporters, all of which are utilized to counteract changes in osmotic pressure (Kuroda et al., 2005; Saum and Müller, 2008).

UV irradiation can lead to both DNA damage and the generation of ROS (Castenholz and Garcia-Pichel, 2012). Strain DJPM01 contained several genes for reducing ROS (e.g., catalase and peroxiredoxin) and for DNA repair, including two photolyases, which may enable photo-repair of DNA during long Antarctic days in the austral summer. UV stress can also be mediated through the production of radiation-absorbing pigments. Strain DJPM01 contains BGCs for prodigiosin and violacein, pigments associated with UV survival in other organisms, such as *Serratia, Streptomyces*, and *Vibrio* species (Borić et al., 2011; Stankovic et al., 2012; Cediel Becerra et al., 2022), and increases the UV-screening ability of commercial sunscreen (Suryawanshi et al., 2015). Violacein has been described in a number of *Massilia* species, including *M. antarctica* from James Ross Island and *M. violaceinigra* from Tianshan glacier, China (Agematu et al., 2011; Myeong et al., 2016; Wang et al., 2018; Yang et al., 2019; Sedláček et al., 2022). To date, prodigiosin has only been described in Antarctic *Massilia* species (Holochová et al., 2020). The prevalence of pigment biosynthesis genes in Antarctic *Massilia* suggests that both violacein and prodigiosin could play a role in adaptation to the environment. However, it remains unclear why only the Antarctic *Massilia* appear to possess genes for prodigiosin. Regardless, climatic change in the MDV likely involves dynamic changes in solar radiation (Obryk et al., 2018), thus the production of UV-screening pigments may not provide a universal advantage to the organisms in question.

In addition to pigments, several other putative secondary metabolite BGCs were identified in the genome, including a homoserine lactone BGC potentially involved in quorum sensing, and a putative siderophore with similarity to myxochelin, shown to play a role in iron acquisition (Kunze et al., 1989; Montgomery et al., 2013). Several RiPP clusters are also present, which produce diverse molecules with a wide variety of functions, including antibacterial or enzyme inhibiting activities (Arnison et al., 2013). Notably, strain DJPM01 contained a putative BGC involved in the biosynthesis of the antifungal polyketide gladiofungin (Niehs et al., 2020). Further analysis of PKS modules in this gene cluster supported its identification as encoding for gladiofungin biosynthetic machinery (Supplementary Figure S4). This is the first report of a *Massilia* with the genetic potential to produce this antifungal. Gladiofungin has been shown to inhibit the fungus *Purpureocillium lilacinum* (Niehs et al., 2020), which has been isolated from the Antarctic peninsula (Gonçalves et al., 2015). The gladiofungin BGC in strain DJPM01 may play a role in inhibition of fungal competitors.

Strain DJPM01 has a similar abundance of genes associated with cold adaptation when compared to other psychrotolerant *Massilia* species, including genes for protein chaperones, RNA helicases, and fatty acid biosynthesis. Minor differences exist between strain DJPM01 and the closely related *M. frigida*, including the presence of specific toxin-antitoxin genes, which are believed to play a role in stress response (Fucich and Chen, 2020). Compared to non-Antarctic *Massilia*, the six *Massilia* isolated from Antarctic regions contained a significantly greater abundance of secondary metabolite BGCs (Supplementary Table S4). The genetic potential to synthesize a wide arsenal of molecules in response to specific stressors under poly-extreme conditions would impart an ecological advantage (Giddings and Newman, 2015b; Sayed et al., 2020). Of the putatively identified BGCs, the myxochelin cluster is present across all six genomes, suggesting a strong selection pressure for iron acquisition (Kunze et al., 1989). Other gene clusters that are only present in one or two genomes, such as cephamycin and the gladiofungin we identified here, may play more specialized roles in competitive inhibition of other community members *in situ*. Secondary metabolism of Antarctic microbes remains poorly characterized and the abundance of putative BGCs that encode for yet uncharacterized molecules may be of interest in the search for novel bioactive compounds (Correa and Abreu, 2020). Extreme environments likely offer an untapped wealth of secondary metabolites that may prove useful in a number of industrial or biomedical applications (Giddings and Newman, 2015a; Sayed et al., 2020).

### 4.3. Prodigiosin, a molecule involved in environmental adaptation

**Figure 8:**
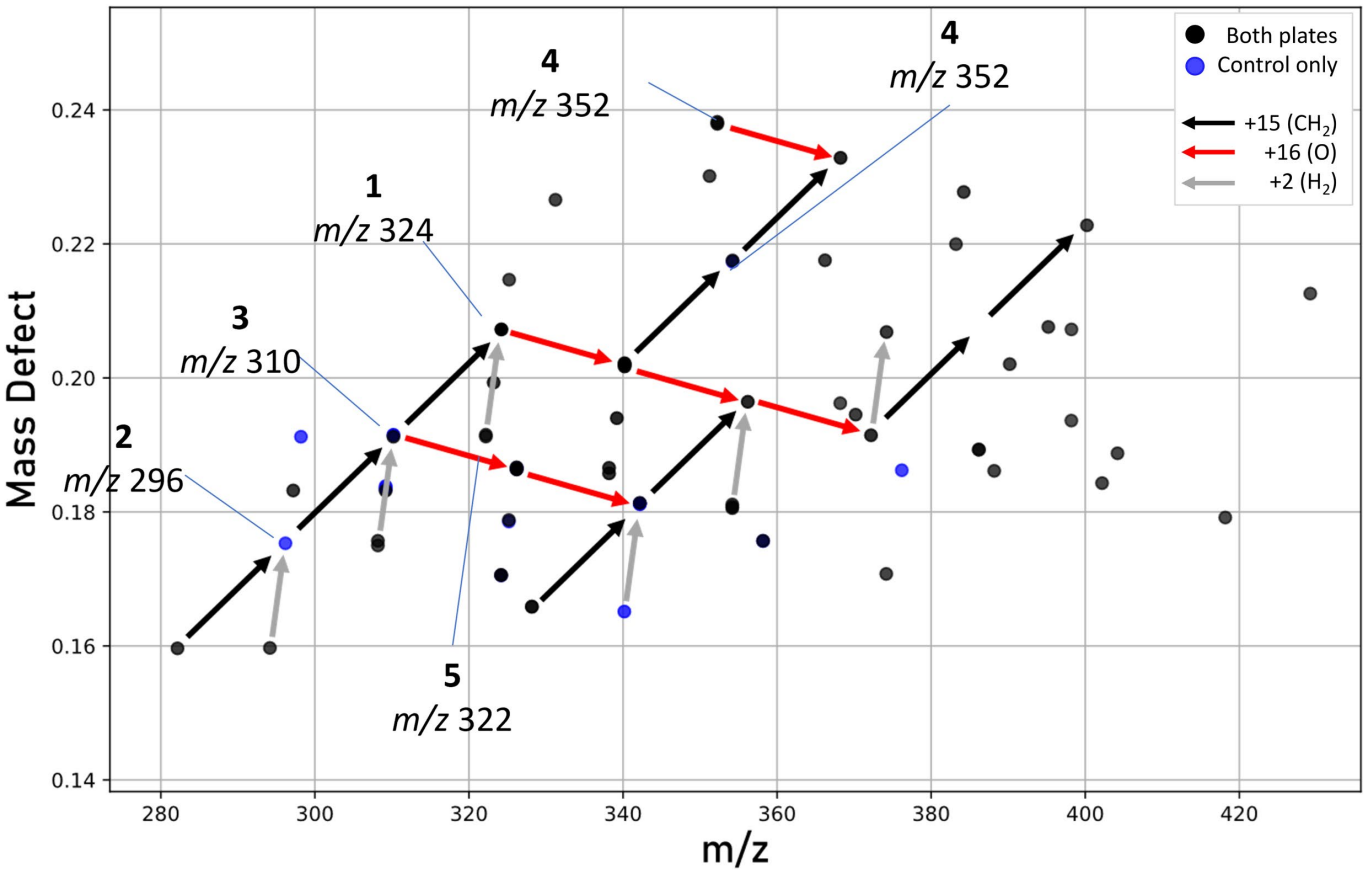
Mass defect plot showing features hypothesized to be prodiginines. Features seen in either both extracts (black dots) or control plate extracts only (blue dots). No unique prodiginine features were seen in UV-treated plates. Arrows represent mass defect vectors, with red denoting the addition of oxygen (+16), black the addition of a methyl group (+15), and gray a loss of hydrogen possibly indicating cyclization.

Our work provides the first molecular characterization of prodiginines produced by a member of the *Massilia* genus. Holochová et al. (2020) noted the pigmentation and BGC for prodigiosin in four Antarctic *Massilia* species, and Woodhams et al. (2018) examined a mesophilic *Massilia* (now *Pseudoduganella*; Lu et al., 2022) for the production of prodigiosins, however none were detected. In our study, mass defect analysis (Figure 8) identified several metabolites congruent with prodiginines detected within similar retention times (5–8 min), including analogs formed from either the addition of oxygen, reduction, or cyclization. Some of these metabolites are known compounds; however, most are unknown and warrant further investigation (He et al., 2022). Due to the high sequence similarity between putative prodigiosin BGCs in *M. frigida*, *M. rubra*, *M. mucilaginosa*, and *M. aquatica*, it is likely that all five red-pigmented Antarctic *Massilia* are genetically capable of producing related pigments.

The biosynthesis of prodigiosin may provide numerous advantages to the cold-adapted *Massilia*, which may serve as pioneer species during microbial mat establishment (Sommers et al., 2019). Given prodigiosin’s role as a UV-protectant in other microbial species (Borić et al., 2011; Stankovic et al., 2012; Cediel Becerra et al., 2022), we sought to determine the effect of intense UV-A radiation on pigment production by strain DJPM01. Following irradiation, we saw a decrease in the abundance of most metabolites, including prodigiosin (1), with the exception of an increase in an oxygenated prodiginine (*m/z* 340.2020), which may represent a UV photolysis product of 2-methyl-3-butyl-prodiginine (3). We did not detect the pigment violacein under any growth condition tested, despite strong genomic evidence for a violacein BGC. Thus, it is likely that this molecule may be produced under yet-unknown conditions, such as in response to predation by metazoans (Choi et al., 2015). The color change (Figure 6C) of UV-treated strain DJPM01 was likely related to prodiginine degradation, as the irradiated prodigiosin standard was observed to lose color intensity following 24-h of UV-A exposure (Supplementary Figure S8). As pigment degradation is likely the result of UV-A exposure, and the rate of photolysis is independent from molecular synthesis, transcriptional or translational response in prodigiosin biosynthesis upon UV exposure should be further investigated. Alternatively, *Massilia* within the natural mat environment may also benefit from other photoprotective mechanisms. Photoprotective compounds can provide a shielding effect to the entire community within microbial mats, so that not only the producers benefit from the UV protection (Gareia-Pichel and Castenholz, 1994).

In addition to UV-protection, prodigiosin has been linked to numerous bioactivities. For example, prodigiosin has been shown to function as an antimicrobial agent against both Gram positive and Gram negative bacteria (Danevčič et al., 2016), and thus may serve as a mediator of interspecies competition within the microbial mat environment. Prodigiosin has also been reported as a nematicidal agent (Habash et al., 2020). Nematodes in the MDV function as grazers within microbial mats, and have been identified as present within the DJP mat (Siegel et al., 1979; Andriuzzi et al., 2018), thus prodigiosin may serve as a defense against overgrazing. Additionally, psychrophilic organisms increase intracellular ATP concentrations to support metabolic activity at low temperatures (Amato and Christner, 2009). In high-density cultures of *S. marcescens*, accumulation of intracellular ATP occurred at a higher rate in prodigiosin pigmented cells, compared to non-pigmented cells (Haddix and Shanks, 2018). Thus, prodigiosin may also play an undescribed role in energetics and adaptation to low temperatures. More directed experiments are necessary to elucidate the ecological function of prodiginines in strain DJPM01 *in situ*.

In the coming decades, changes in solar radiation will impact MDV communities through both direct and indirect processes. The low albedo of MDV soils leads to increased levels of energy absorption compared to the surrounding ice (Dana et al., 1998) resulting in higher summer *in situ* temperatures (Fountain et al., 2014), which could lead to possible hydrological and geochemical changes, such as enhanced permafrost melting and salt mobilization (Ball and Virginia, 2012). Additionally, both anthropogenic and natural (i.e., volcanoes and wildfire) emissions drive variations in solar flux in the MDV, potentially resulting in a decrease UV radiation (Obryk et al., 2018). Thus, microbial community response to UV irradiation will likely vary with local climatic trends. Investigating the responses of individual community members to simulated stressors (i.e., UV radiation) can inform how natural microbial communities may respond to environmental variation associated with anthropogenic climate change.

## 5. Conclusion

Our characterization of *Massilia frigida* strain DJPM01 provides insight into the complex microbial interactions occurring in an Antarctic microbial mat and suggests multiple avenues for future research. In Antarctic *Massilia*, both pigment production and the high number of BGCs suggests *Massilia* may play a role in mediating microbial interactions in Antarctic ecosystems and highlights the importance of investigating Antarctic microbial isolates for their production of bioactive compounds. Prodigiosin has been implicated in bactericidal, nematocidal, and UV-screening activities, though the role it plays *in situ* in complex, multi-species Antarctic microbial mats warrants further investigation. Our data demonstrate that UV radiation impacts the accumulation of this pigment in *Massilia*, thus the abundance of this molecule may be influenced by increased variation in solar radiation. The changing climate in the MDV system is complex with effects on microbial communities uncertain; understanding the role of secondary metabolites in the MDV communities, especially under ecologically relevant conditions can inform how these communities will respond to environmental change.

## Data availability statement

The datasets presented in this study can be found in online repositories. The names of the repository/repositories and accession number(s) can be found at: https://www.ncbi.nlm.nih.gov/genbank/, JAQOZV000000000. https://www.ncbi.nlm.nih.gov/genbank/, OQ346149.

## Author contributions

JAM and L-AG conceived of the project and acquired funding and resources. JAM collected and processed the samples. JS, L-AG, RS, and JAM contributed to the design of the experiments. JS performed the culture work, DNA extraction, genomic analyses, and wrote the first draft of the manuscript. JS and L-AG extracted samples for metabolomics. RS analyzed the metabolite samples. L-AG and RS interpreted the metabolomics data. All authors contributed to the article and approved the submitted version.

## Funding

This work was funded by the National Science Foundation OPP #2148730 and OPP #1643687 (to JAM) and OPP #2148731 (to L-AG) with additional support from the University of Tennessee Office of Research, Innovation and Economic Development (to JAM).

## Conflict of interest

The authors declare that the research was conducted in the absence of any commercial or financial relationships that could be construed as a potential conflict of interest.

## Publisher’s note

All claims expressed in this article are solely those of the authors and do not necessarily represent those of their affiliated organizations, or those of the publisher, the editors and the reviewers. Any product that may be evaluated in this article, or claim that may be made by its manufacturer, is not guaranteed or endorsed by the publisher.

## Footnotes

^1^ https://lpsn.dsmz.de/genus/massilia

^2^ https://proksee.ca/
