# Supplementary material for: Genomic and phenotypic characterization of a red-pigmented strain of Massilia frigida isolated from an Antarctic microbial mat
Source: Front Microbiol. 2023 May 12;14:1156033. doi: 10.3389/fmicb.2023.1156033 (PMC10213415; doi:10.3389/fmicb.2023.1156033)
Supplement: Supplementary file 1 [file Data_Sheet_1.docx]

Supplementary Material

Genomic and phenotypic characterization of a red-pigmented strain of *Massilia frigida* isolated from an Antarctic microbial mat

Jacob M. C. Shaffer^1^, Lesley-Ann Giddings^2^, Robert M. Samples^2^, Jill A. Mikucki^1^*

*** Correspondence:** Jill A. Mikucki: jmikucki@utk.edu

# Supplementary Data

## Pigment extraction for UV-Vis analysis.

## Pigments were extracted from individual colonies of strain DJPM01 following mechanical cell lysis using a FastPrep-24 bead beating system (MP Bio). Cells were incubated for 10 minutes with a 10% SDS solution (5% final concentration) before the addition of water-saturated butanol. Cells were centrifuged for 20 minutes at 14,000 x g and the absorption spectrum of the organic phase was analyzed from 400-700 nm in steps of 5 nm using a Synergy 2 Plate Reader (Biotek; Winooski, VT).

## Metabolomics data processing, filtering, and statistical analyses

# The peak list and fragment database were exported from Progenesis and analyzed with MPACT (Samples et al., 2022). Data were filtered using solvent blank, mispicked peak, reproducibility, and in-source fragment filters. Blank filtering using EtOAc blanks was applied based on a relative group parsing threshold of 0.01. A minimum reproducibility threshold of 0.5 median coefficient of variation (CV) among technical replicates was applied. The presence or absence of features in a group of samples was determined using a relative abundance threshold of 0.05 compared to the sample group in which a feature was most abundant. This threshold was applied during blank filtering to remove features whose abundance in solvent blanks was greater than 5% of their abundance in experimental samples. In-source ion filtering and mispicked peak filtering were disabled.

## Supplementary References

Blin, K., Shaw, S., Kloosterman, A. M., Charlop-Powers, Z., van Wezel, G. P., Medema, M. H., et al. (2021). antiSMASH 6.0: improving cluster detection and comparison capabilities. *Nucleic Acids Research* 49, W29–W35. doi: 10.1093/nar/gkab335.

Kanehisa, M., Sato, Y., and Morishima, K. (2016). BlastKOALA and GhostKOALA: KEGG Tools for Functional Characterization of Genome and Metagenome Sequences. *Journal of Molecular Biology* 428, 726–731. doi: 10.1016/j.jmb.2015.11.006.

Meier-Kolthoff, J. P., Carbasse, J. S., Peinado-Olarte, R. L., and Göker, M. (2022). TYGS and LPSN: a database tandem for fast and reliable genome-based classification and nomenclature of prokaryotes. *Nucleic Acids Research* 50, D801–D807. doi: 10.1093/nar/gkab902.

Rodriguez-R, L. M., and Konstantinidis, K. T. (2016). The enveomics collection: a toolbox for specialized analyses of microbial genomes and metagenomes. PeerJ Preprints doi: 10.7287/peerj.preprints.1900v1 (Accessed September 12, 2022).

Samples, R., Puckett, S., and Balunas, M. (2022). MPACT: An advanced informatics tool for metabolomics and data visualization of specialized metabolites from complex microbial samples. *ChemRxiv* [Preprint]. Available at: https://doi.org/10.26434/chemrxiv-2022-r0xbx (Accessed January 22, 2023).

Seemann, T. (2014). Prokka: rapid prokaryotic genome annotation. *Bioinformatics* 30, 2068–2069. doi: 10.1093/bioinformatics/btu153.

Skinnider, M. A., Johnston, C. W., Gunabalasingam, M., Merwin, N. J., Kieliszek, A. M., MacLellan, R. J., et al. (2020). Comprehensive prediction of secondary metabolite structure and biological activity from microbial genome sequences. *Nat Commun* 11, 6058. doi: 10.1038/s41467-020-19986-1.

# Supplementary Figures and Tables

## Supplementary Figures


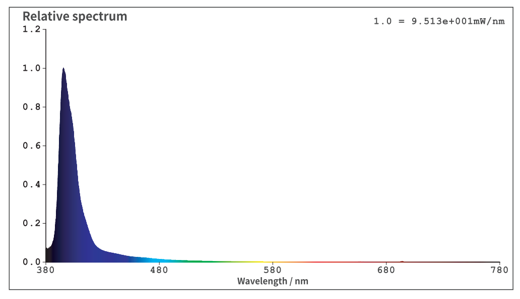


**Supplementary Figure 1.** Output spectrum of UV-A light, as sourced from the Item User Guide (SANSI Lighting 5W LED Black Light Bulb; Item Model Number C21BB-TE26-5W-UVA).


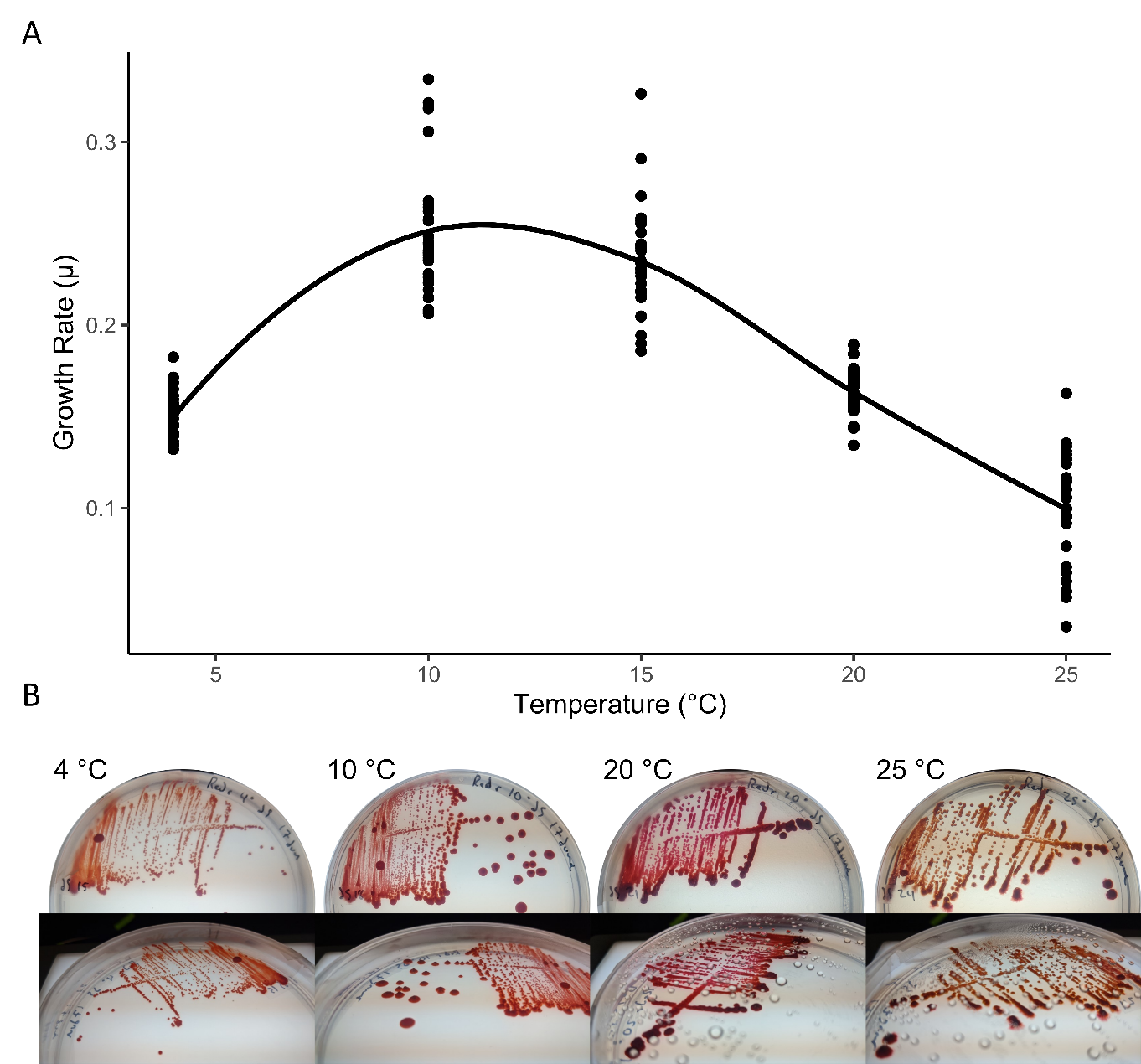


**Supplementary Figure 2.** (A) Calculated growth rates for strain DJPM01 grown at temperatures 4, 10, 15, 20, and 25 °C. Growth curves were performed in a 96-well plate and measurements were taken once per day. Wells on the outside of the plate were excluded from final calculations (n=30). (B) Images of streak plates of strain DJPM01 incubated at 4, 10, 20, and 25 °C, respectively.


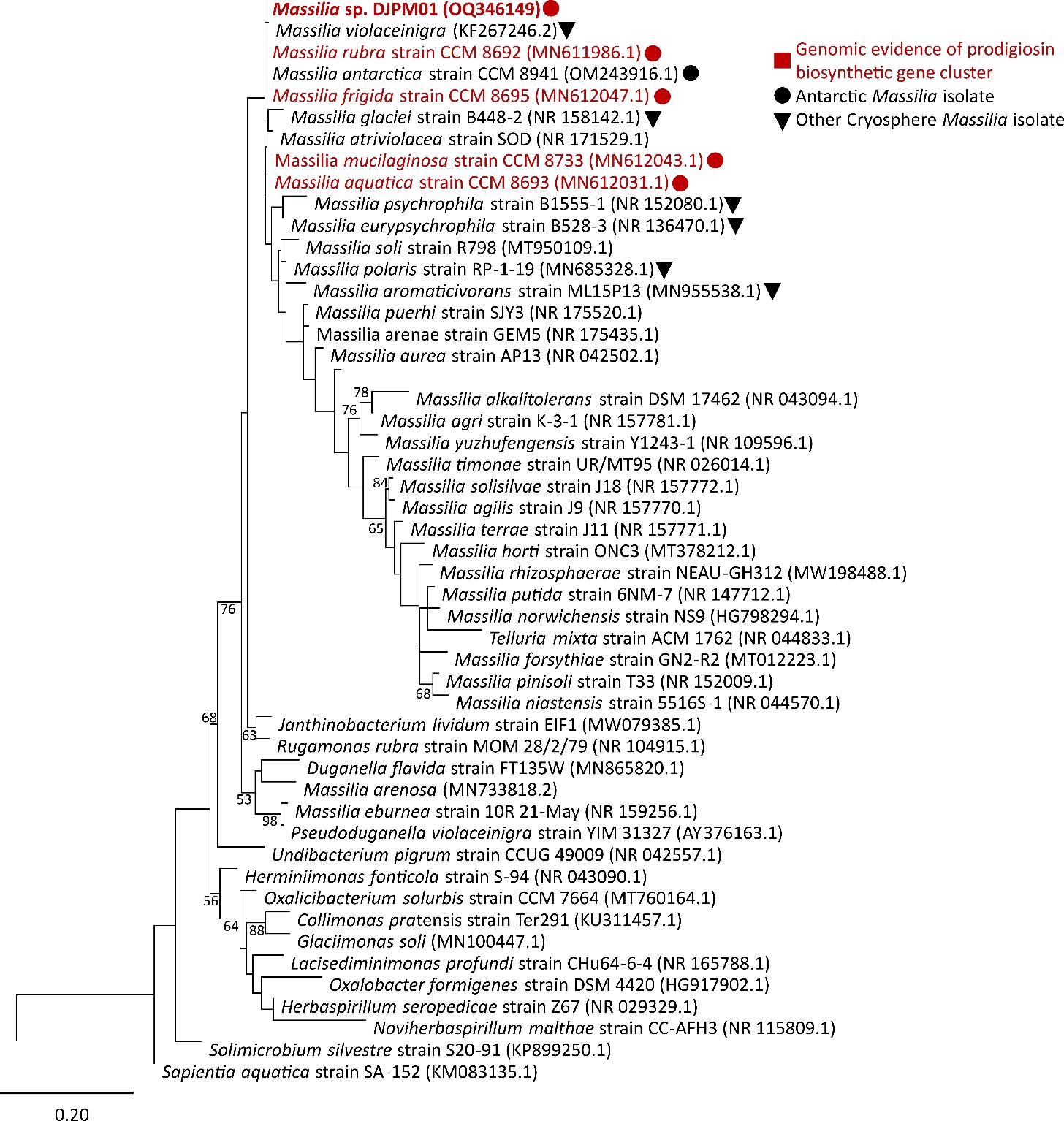


**Supplementary Figure 3.** Maximum likelihood phylogenetic tree constructed using 16S rRNA genes from members of the Oxalobacteraceae family and strain DJPM01. Sequences from strains with phenotypic and genotypic evidence for prodigiosin production are indicated in red. Strain DJPM01 was highly similar to other Antarctic and cryosphere *Massilia* isolates. Bootstrap values >50 (9999 replicates) are indicated at nodes. An Antarctic *Shewanella* species (strain BF02_Schw) was used as an outgroup (DQ677870.1).


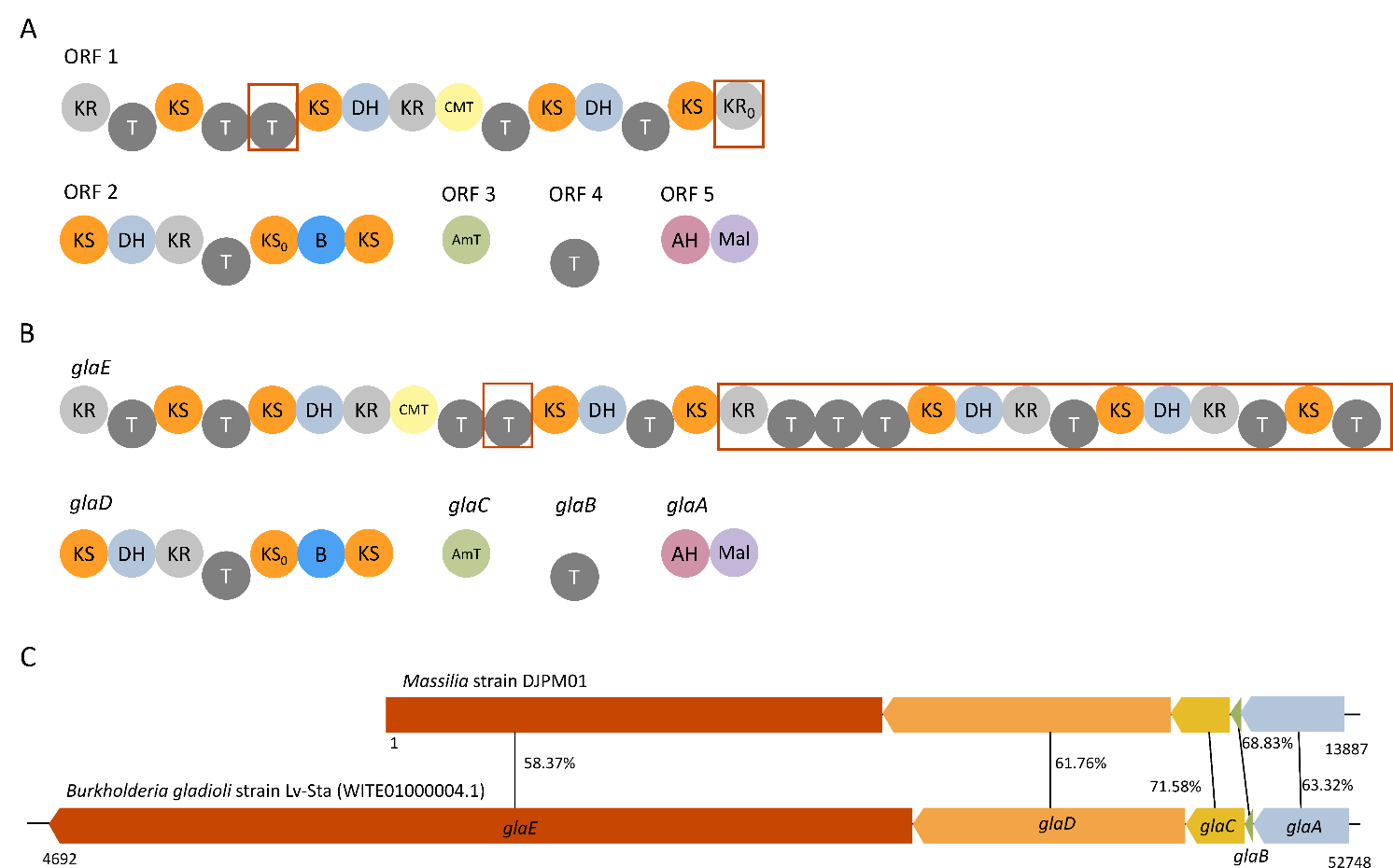


**Supplementary Figure 4.** PRISM v4.4.5 **(Skinnider et al., 2020)** output for (A) putative iso-migrastatin BGC in strain DJPM01 genome and (B) gladiofungin BGC in *Burkholderia gladioli* strain Lv-Sta (WITE01000004.1), with missing PKS modules boxed in red. (C) Alignment of BGCs, highlighting similarity between individual genes. *AH - acylhydrolase. AmT - aminotransferase. B - branching domain. CMT - C-methyltransferase. DH - dehydrotase. KR - ketoreductase. KR0 - inactive ketoreductase. KS - ketosynthase. Mal - acyltransferase (malonyl-CoA). T - Thiolation.*

**
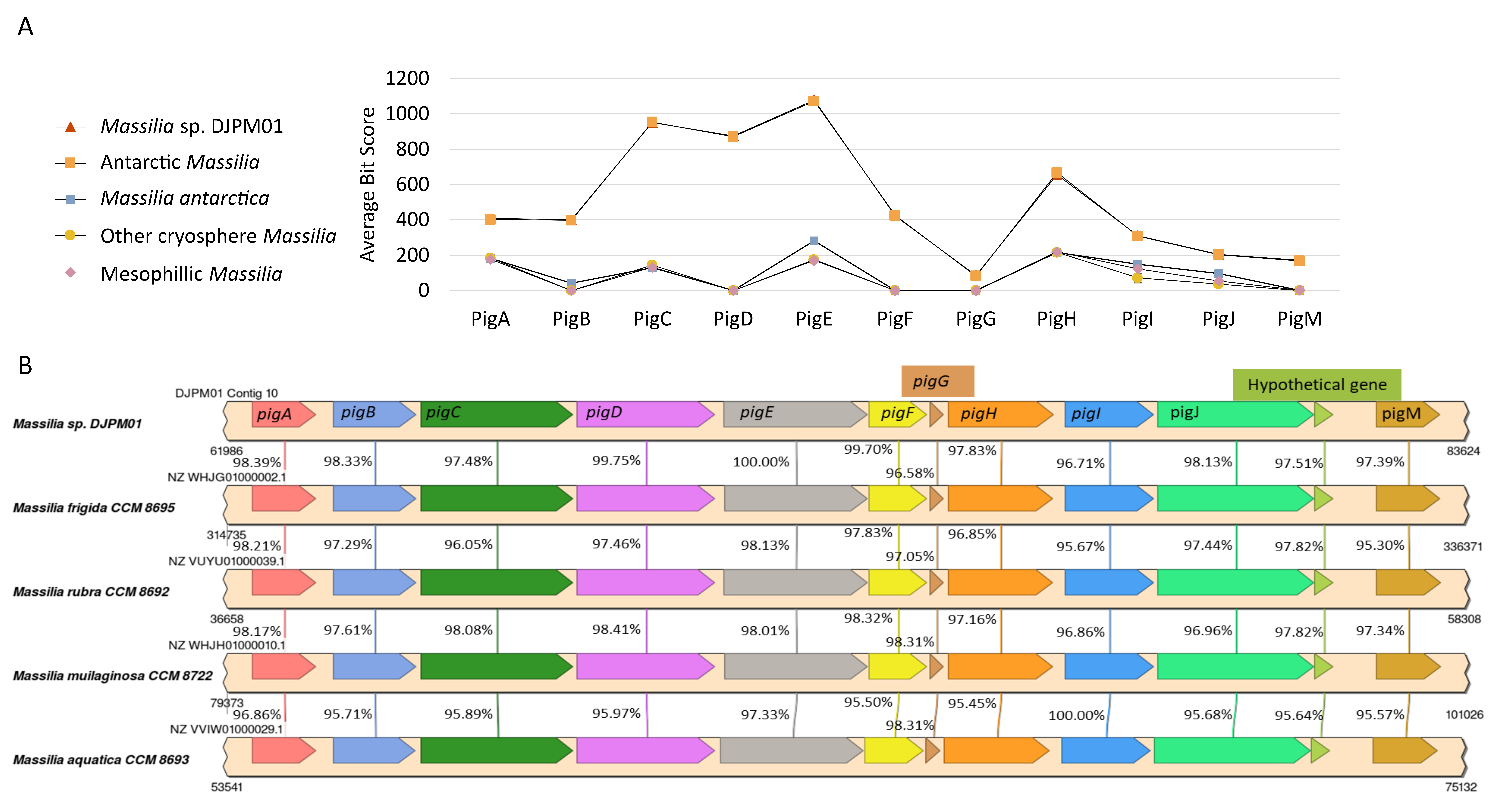
**

**Supplementary Figure 5.** (A) Summary of sequence comparisons between *Serratia* sp. strain ATCC 39006 and RefSeq *Massilia* genomes. Similarities are expressed in Average bit score for each gene per group of genomes. (B) Comparison of putative prodigiosin BGCs for Antarctic *Massilia* species. Lines between individual genes highlight percent identity between those two genes.


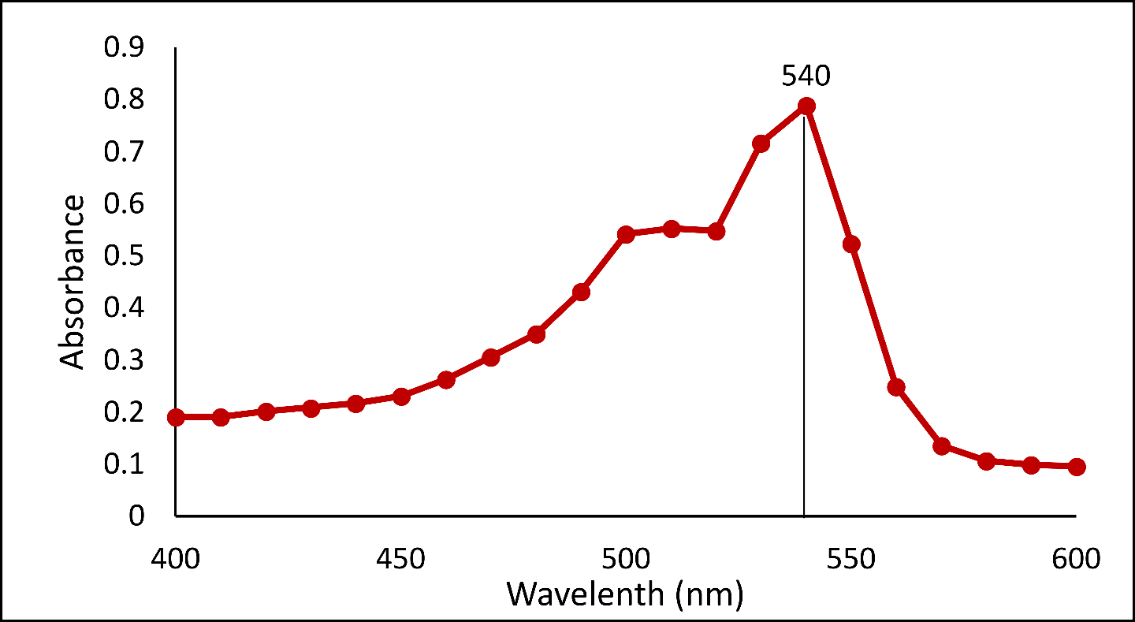


**Supplementary Figure 6.** Absorbance spectrum of extracted pigment from colonies of strain DJPM01 in butanol. The absorbance maximum is at 540 nm, which corresponds to literature values for prodigiosin at acidic pH.

**
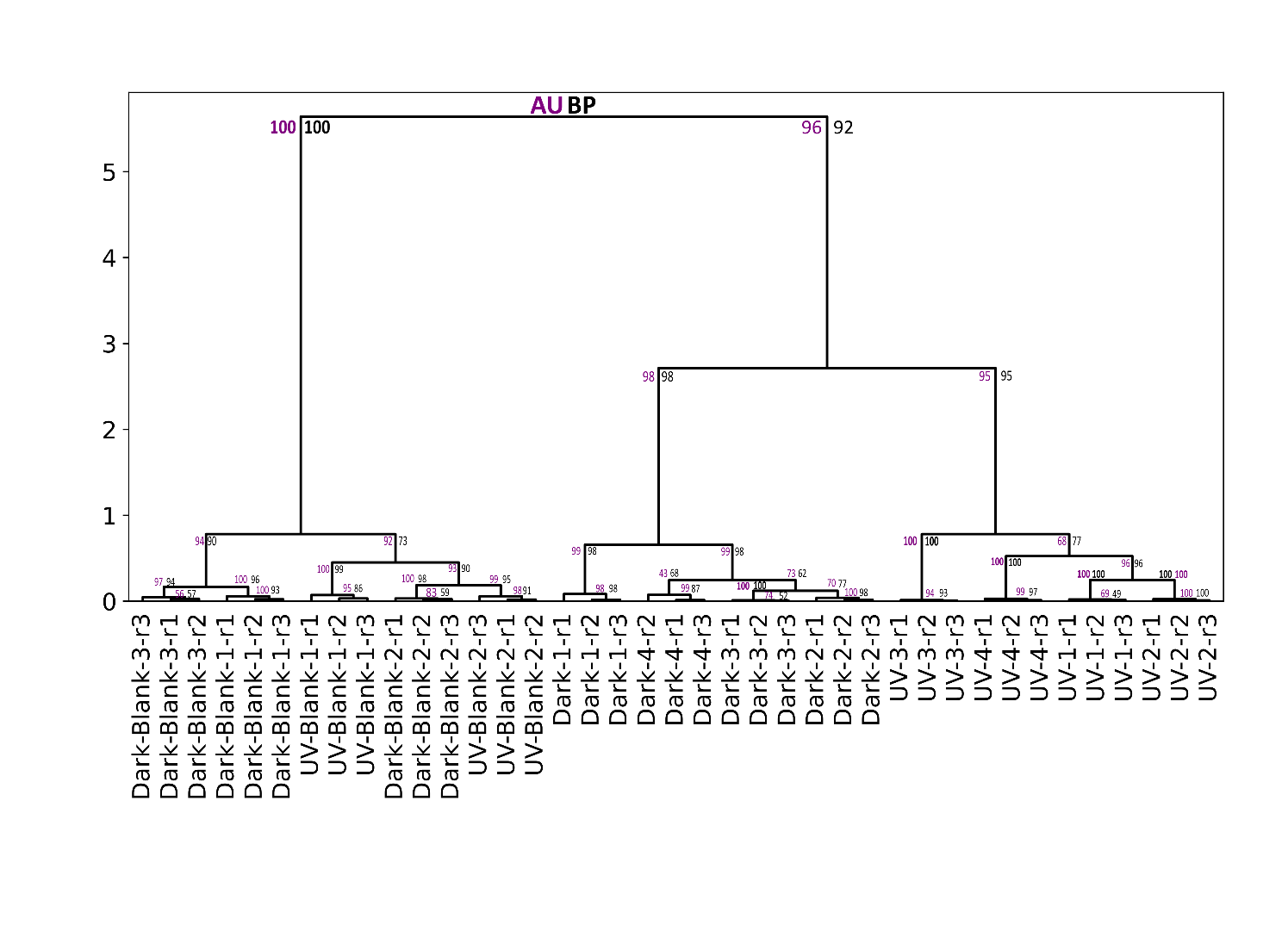
**

**Supplementary Figure 7.** Statistically significant differences between blanks, controls, and UV-treated samples. Hierarchical clustering analysis of filtered LC/MS data demonstrating the statistically significant differences between samples (4 biological replicates injected in triplicate). Media blanks, UV-treated DJPM01 on R2A, and DJPM01 on R2A controls are indicated by black, red, and blue, respectively.

**
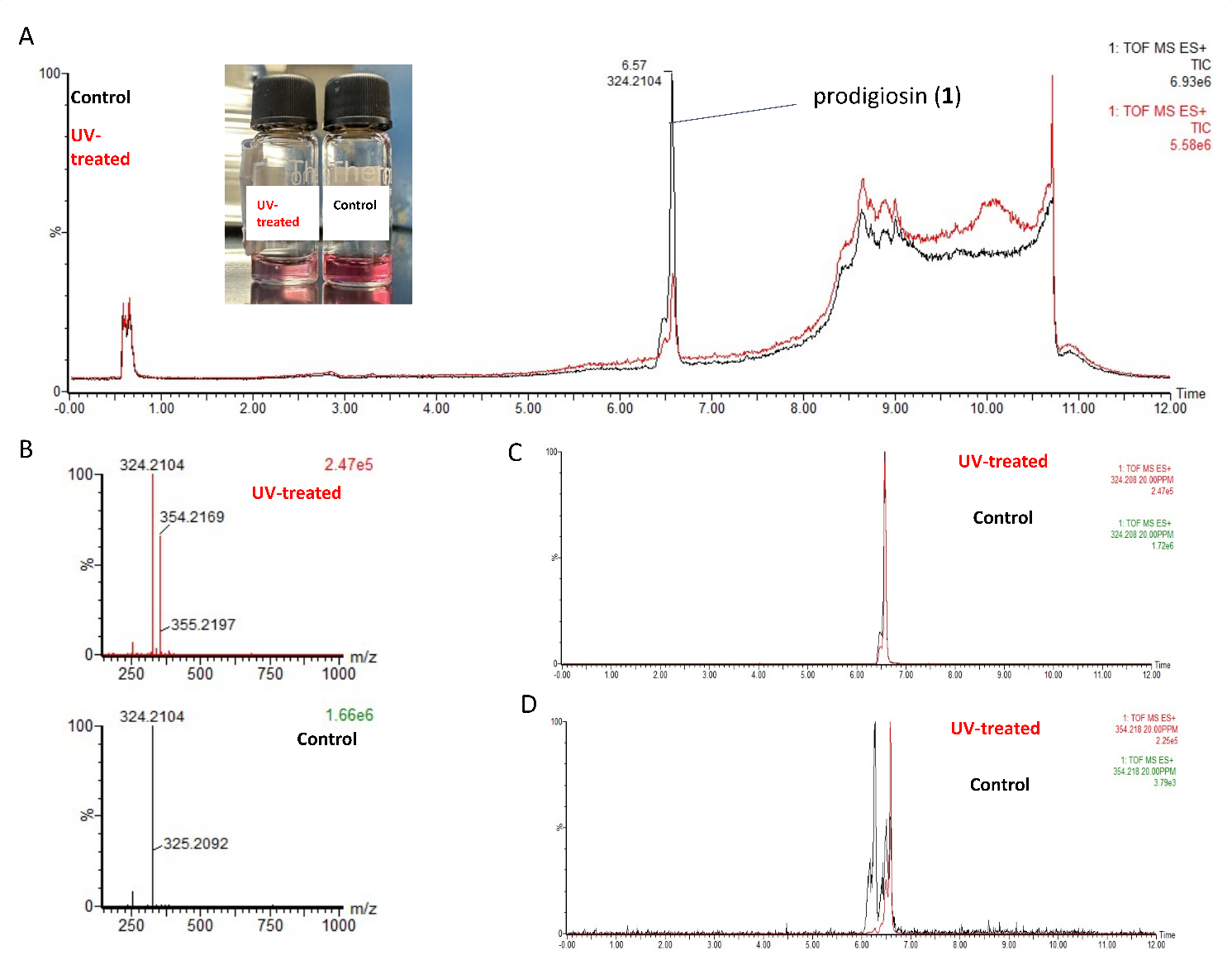
**

**Supplementary Figure 8.** UV Photolysis of prodigiosin standard. (A) TIC chromatograms of prodigiosin standard (black) and UV-exposed prodigiosin standard (red) Prodigiosin is present at 6.57 minutes. Inset: Image of degraded standard (left) compared to control standard (right). (B) MS1 spectra at 6.57 minutes for UV-exposed (black) and unexposed (red) prodigiosin standards showing the appearance of a coeluting photolysis product with m/z 354.2169. (C) EIC for prodigiosin (324.208 m/z) in UV-exposed (black) and unexposed (red) prodigiosin standards showing decreased prodigiosin content. (D) EIC for a prodigiosin photolysis product featuring addition of CH2O (324.208 m/z) in UV-exposed (black) and unexposed (red) prodigiosin standards showing an approximate 100-fold increase in abundance of the photolysis product with tR of 6.59 minutes.

**Supplementary Table 1.** RefSeq *Massilia* genomes used in comparative analysis. Average nucleotide identity was calculated using the enviomics collection **(Rodriguez-R and Konstantinidis, 2016)** and digital DNA-DNA hybridization was performed using Genome-to-Genome Distance Calculator v3.0 **(Meier-Kolthoff et al., 2022)**.

| **RefSeq Genome (Accession Number)** | **Isolation Site** | **Comparison to strain DJPM01** | |
| --- | --- | --- | --- |
|  |  | Average Nucleotide Identity (%) | Digital DNA-DNA Hybridization [model C.I.] (%) |
| *Massilia agilis* JCM 31605 (GCF 024756255) | Forest soil; Suwon, South Korea | 79.41 | 21.1 [18.9 - 23.6} |
| *Massilia agri* JCM 31661 (GCF 024753255) | Grassland Soil; Morang, Nepal | 79.02 | 20.6 [18.4 - 23] |
| *Massilia alkalitolerans* DSM 17462 (GCF 000427785) | Soil; Yunnan Province China | 79.25 | 20.8 [18.6 - 23.2] |
| *Massilia antarctica* P9640 (GCF 015689335) | Brook water; James Ross Island, Antarctica | 91.92 | 46.5 [44 - 49.1] |
| *Massilia aquatica* CCM 8693 (GCF 011682045) | Small stones; James Ross Island, Antarctica | 87.94 | 34.4 [31.9 - 36.9] |
| *Massilia arenae* GEM5 (GCF 008014745) | Sand; Qinghai Province, China | 79.06 | 20.5 [18.3 - 23] |
| *Massilia arenosa* MC02 (GCF 004614185.1) | Sandy-loam maize soil; United States | 79.07 | 20.6 [18.4 - 23] |
| *Massilia aromaticivorans* ML15P13 (GCF 013003915) | Arctic Soil; Svalbard, Norway | 79.19 | 21.1 [18.8 - 23.5] |
| *Massilia atriviolacea* SOD (GCF 003953935) | Soil; Hefei, China | 88.04 | 34.5 [32.1 - 37] |
| *Massilia aurea* JCM 13879 (GCF 024753205) | Drinking water distribution system; Seville, Spain | 78.92 | 20.3 [18.1 - 22.7] |
| *Massilia cavernae* K1S02-61 (GCF 003590855) | Soil from karst cave; Guizhou, China | 81.25 | 23.2 [20.9 - 25.7] |
| *Massilia eburnea* JCM 31587 (GCF 009720745) | Lagoon sediments; Japan | 78.53 | 20 [17.8 - 22.4] |
| *Massilia eurypsychrophila* JCM 30074 (GCF 002760655) | Ice core; Muztagh Glacier, Xinjiang, China | 81.41 | 23.1 [20.8 - 25.6] |
| *Massilia forsythiae* GN2-R2 (GCF 012849555) | Flower of *Forsythia koreana*; Gongju-si, South Korea | 79.47 | 21.5 [19.3 - 24] |
| *Massilia frigida* CCM 8695 (GCF 011682175) | Water; James Ross Island, Antarctica | 97.36 | 75.6 [72.6 - 78.4] |
| *Massilia glaciei* B448-2 (GCF 003011895) | Thawed water; Muztagh Glacier, Xinjiang, China | 81.21 | 23.2 [21 - 25.7] |
| *Massilia horti* ONC3 (GCF 004614195) | Garden soil subsurface; United States | 79.39 | 21.2 [19 - 23.6] |
| *Massilia mucilaginosa* CCM 8733 (GCF 011682145) | Water; James Ross Island, Antarctica | 88.41 | 35.2 [32.8 - 37.7] |
| *Massilia niastensis* DSM 21313 (GCF 000382345) | Air Sample; Suwon, South Korea | 79.88 | 21.4 [19.2 - 23.9] |
| *Massilia norwichensis* LMG 28164 (GCF 024753245) | Air sample; Norwich, United Kingdom | 79.20 | 20.8 [18.6 - 23.3] |
| *Massilia oculi* CCUG 43427 (GCF 003143515) | Human eye; Linköping, Sweden | 79.10 | 20.8 [18.6 - 23.3] |
| *Massilia pinisoli* JCM 31316 (GCF 024753285) | Forest soil; Suwon, South Korea | 78.55 | 20.4 [18.1 - 22.8] |
| *Massilia polaris* RP-1-19 (GCF 012927275) | Arctic Soil; Svalbard, Norway | 81.30 | 22.9 [20.6 - 25.4] |
| *Massilia psychrophila* JCM 30813 (GCF 002760665) | Ice core; Muztagh Glacier, Xinjiang, China | 80.81 | 22.4 [20.2 - 24.9] |
| *Massilia puerhi* SJY3 (GCF 009861485) | Soil; China | 79.41 | 20.7 [18.5 - 23.2] |
| *Massilia putida* 6NM-7 (GCF 001941825) | Wolfram mining tailing; Jiangxi, China | 78.81 | 20.9 [18.7 - 23.4] |
| *Massilia rhizosphaerae* NEAU-GH312 (GCF 016093545) | Soil; Heilongjiang, China | 78.65 | 20.4 [18.2 - 22.8] |
| *Massilia rubra* CCM 8692 (GCF 011682065) | Stone; James Ross Island, Antarctica | 88.31 | 35.1 [32.6 - 37.6] |
| *Massilia soli* R798 (GCF 016809835) | Soil; South Korea | 81.09 | 22.8 [20.5 - 25.2] |
| *Massilia solisilvae* JCM 31607 (GCF 024753215) | Forest soil; Suwon, South Korea | 79.47 | 21.2 [18.9 - 23.6] |
| *Massilia terrae* JCM 31606 (GCF 024753145) | Forest soil; Suwon, South Korea | 79.42 | 21.3 [19.1 - 23.8] |
| *Massilia timonae* NEU (GCF 001866515) | Forest soil; Neuchatel, Switzerland | 79.26 | 21 [18.7 - 23.4] |
| *Massilia violaceinigra* B2 (GCF 002752675) | Glacial permafrost;Tianshan Glacier, Xinjian, China | 91.38 | 44.5 [42 - 47.1] |
| *Massilia yuzhufengensis* CGMCC 1.12041 (GCF 900112225) | Ice core;  Yuzhufeng Glacier, China | 79.29 | 21 [18.8 - 23.4] |

**Supplementary Table 2.** Summary of genomic adaptations present in DJPM01. Coding sequences were identified using Prokka v1.13.7 **(Seemann, 2014)** and KEGG identities were provided using GhostKOALA v2.2 **(Kanehisa et al., 2016)**

| **Gene Product name** | **Sequence ID** | **KO** | **GhostKOALA Annotation** | **Score** |
| --- | --- | --- | --- | --- |
| **Cold stress** |  |  |  |  |
| Cold Shock Protein CspA | HPNLJDEO_02266 | K03704 | cspA; cold shock protein | 135 |
| Cold Shock Protein CspA | HPNLJDEO_03891 | K03704 | cspA; cold shock protein | 138 |
| ATP-dependent Clp protease | HPNLJDEO_00228 | K03695 | clpB; ATP-dependent Clp protease ATP-binding subunit ClpB | 1410 |
| ATP-dependent Clp protease | HPNLJDEO_05808 | K03695 | clpB; ATP-dependent Clp protease ATP-binding subunit ClpB | 310 |
| molecular chaperone DnaJ | HPNLJDEO_04280 | K03686 | dnaJ; molecular chaperone DnaJ | 62 |
| molecular chaperone DnaJ | HPNLJDEO_01662 | K03686 | dnaJ; molecular chaperone DnaJ | 608 |
| molecular chaperone DnaK | HPNLJDEO_01663 | K04043 | dnaK, HSPA9; molecular chaperone DnaK | 1087 |
| molecular chaperone DnaK | HPNLJDEO_01779 | K04043 | dnaK, HSPA9; molecular chaperone DnaK | 876 |
| molecular chaperone DnaK | HPNLJDEO_05795 | K04043 | dnaK, HSPA9; molecular chaperone DnaK | 1014 |
| chaperonin GroEL | HPNLJDEO_05678 | K04077 | groEL, HSPD1; chaperonin GroEL [EC:5.6.1.7] | 903 |
| chaperonin GroES | HPNLJDEO_05679 | K04078 | groES, HSPE1; chaperonin GroES | 169 |
| molecular chaperone GrpE | HPNLJDEO_01664 | K03687 | GRPE; molecular chaperone GrpE | 249 |
| stearoyl-CoA desaturase | HPNLJDEO_04730 | K00507 | SCD, desC; stearoyl-CoA desaturase (Delta-9 desaturase) [EC:1.14.19.1] | 662 |
| acyl-CoA thioesterase TesA | HPNLJDEO_03409 | K10804 | tesA; acyl-CoA thioesterase I [EC:3.1.2.- 3.1.2.2 3.1.1.2 3.1.1.5] | 261 |
| acyl-CoA thioesterase YciA | HPNLJDEO_02203 | K10806 | yciA; acyl-CoA thioesterase YciA [EC:3.1.2.-] | 224 |
| ATP-dependent RNA helicase DbpA | HPNLJDEO_00246 | K05591 | dbpA; ATP-dependent RNA helicase DbpA [EC:3.6.4.13] | 707 |
| ATP-dependent RNA helicase DeaD | HPNLJDEO_05195 | K05592 | deaD, cshA; ATP-dependent RNA helicase DeaD [EC:3.6.4.13] | 845 |
| ATP-dependent RNA helicase RhlE | HPNLJDEO_01449 | K11927 | rhlE; ATP-dependent RNA helicase RhlE [EC:3.6.4.13] | 573 |
| ATP-dependent RNA helicase RhlE | HPNLJDEO_02635 | K11927 | rhlE; ATP-dependent RNA helicase RhlE [EC:3.6.4.13] | 657 |
| ATP-dependent RNA helicase RhlE | HPNLJDEO_02720 | K11927 | rhlE; ATP-dependent RNA helicase RhlE [EC:3.6.4.13] | 634 |
| **DNA Photorepair** |  |  |  |  |
| deoxyribodipyrimidine photolyase phr | HPNLJDEO_05006 | K01669 | phr, PHR1; deoxyribodipyrimidine photo-lyase [EC:4.1.99.3] | 655 |
| (6-4)DNA photolyase phrB | HPNLJDEO_04024 | K06876 | phrB; (6-4)DNA photolyase [EC:4.1.99.13] | 434 |
| **Oxidative Stress** |  |  |  |  |
| catalase | HPNLJDEO_00224 | K03781 | katE, CAT, catB, srpA; catalase [EC:1.11.1.6] | 737 |
| catalase | HPNLJDEO_02565 | K03781 | katE, CAT, catB, srpA; catalase [EC:1.11.1.6] | 1056 |
| catalase | HPNLJDEO_04366 | K03781 | katE, CAT, catB, srpA; catalase [EC:1.11.1.6] | 677 |
| catalase | HPNLJDEO_04511 | K03781 | katE, CAT, catB, srpA; catalase [EC:1.11.1.6] | 66 |
| catalase | HPNLJDEO_04578 | K03781 | katE, CAT, catB, srpA; catalase [EC:1.11.1.6] | 783 |
| catalase | HPNLJDEO_04622 | K03781 | katE, CAT, catB, srpA; catalase [EC:1.11.1.6] | 717 |
| lipoyl-dependent peroxiredoxin | HPNLJDEO_01672 | K04063 | osmC, ohr; lipoyl-dependent peroxiredoxin [EC:1.11.1.28] | 189 |
| lipoyl-dependent peroxiredoxin | HPNLJDEO_04258 | K04063 | osmC, ohr; lipoyl-dependent peroxiredoxin [EC:1.11.1.28] | 191 |
| superoxide dismutase | HPNLJDEO_03214 | K04564 | SOD2; superoxide dismutase, Fe-Mn family [EC:1.15.1.1] | 358 |
| **Osmotic Stress** |  |  |  |  |
| sodium/hydrogen antiporter (NHA1) | HPNLJDEO_02400 | K24160 | NHA1, SOD2; sodium/hydrogen antiporter | 509 |
| Na+:H+ antiporter (NhaA) | HPNLJDEO_04494 | K03313 | nhaA; Na+:H+ antiporter, NhaA family | 520 |
| monovalent cation:H+ antiporter | HPNLJDEO_05830 | K03316 | TC.CPA1; monovalent cation:H+ antiporter, CPA1 family | 493 |
| citrate synthase | HPNLJDEO_05492 | K01647 | CS, gltA; citrate synthase [EC:2.3.3.1] | 656 |
| glutamate synthase – large chain | HPNLJDEO_05872 | K00265 | gltB; glutamate synthase (NADPH) large chain [EC:1.4.1.13] | 289 |
| glutamate synthase – small chain | HPNLJDEO_05871 | K00266 | gltD; glutamate synthase (NADPH) small chain [EC:1.4.1.13] | 637 |
| glutamate/aspartate transport system substrate-binding protein | HPNLJDEO_05974 | K10001 | gltI, aatJ; glutamate/aspartate transport system substrate-binding protein | 791 |
| glutamate/aspartate transport system permease protein | HPNLJDEO_05973 | K10003 | gltJ, aatQ; glutamate/aspartate transport system permease protein | 2712 |
| glutamate/aspartate transport system permease protein | HPNLJDEO_05972 | K10002 | gltK, aatM; glutamate/aspartate transport system permease protein | 854 |
| glutamate/aspartate transport system ATP-binding protein | HPNLJDEO_02196 | K10004 | gltL, aatP; glutamate/aspartate transport system ATP-binding protein [EC:7.4.2.1] | 373 |
| glutamate/aspartate transport system ATP-binding protein | HPNLJDEO_05971 | K10004 | gltL, aatP; glutamate/aspartate transport system ATP-binding protein [EC:7.4.2.1] | 338 |
| formimidoylglutamate deiminase | HPNLJDEO_01030 | K05603 | hutF; formimidoylglutamate deiminase [EC:3.5.3.13] | 320 |
| N-formylglutamate deformylase | HPNLJDEO_01029 | K01458 | hutG; N-formylglutamate deformylase [EC:3.5.1.68] | 401 |
| histidine ammonia-lyase | HPNLJDEO_06057 | K01745 | hutH, HAL; histidine ammonia-lyase [EC:4.3.1.3] | 422 |
| imidazolonepropionase | HPNLJDEO_01032 | K01468 | hutI, AMDHD1; imidazolonepropionase [EC:3.5.2.7] | 554 |
| urocanate hydratase | HPNLJDEO_01027 | K01712 | hutU, UROC1; urocanate hydratase [EC:4.2.1.49] | 350 |

**Supplementary Table 3.** antiSMASH v. 6.0.0 **(Blin et al., 2021)** output table for strain DJPM01.

| **antiSMASH Category** | **Contig** | **Position** | **Most similar known**  **cluster (% Similarity)** |
| --- | --- | --- | --- |
| Acyl Amino Acids | 2 | 208027 – 272514 |  |
| Acyl Amino Acids, RiPP-like | 38 | 1 – 34955 |  |
| Arylpolyene | 77 | 1 – 27333 | APE Ec (36%) |
| Homoserine Lactone | 16 | 98016 – 118603 |  |
| Indole, TransAT-PKS, NRP | 12 | 2347 – 102882 | Violacein (100%) |
| Lanthipeptide Class iv | 4 | 13765 – 36443 |  |
| Lanthipeptide Class-iv | 6 | 1 – 14584 |  |
| NRPS | 28 | 50388 – 91004 | Cephamycin C (36%) |
| NRPS | 36 | 1 – 37992 | Myxochelin (41%) |
| NRPS-like | 96 | 1 – 11867 |  |
| Prodigiosin | 10 | 53690 – 90993 | Prodigiosin (42%) |
| RiPP-like | 123 | 1 – 4714 |  |
| RiPP-like | 29 | 67380 – 78210 |  |
| RiPP-like | 53 | 31685 – 43283 |  |
| Terpene | 62 | 26592 – 43142 |  |
| TransAT-PKS | 3 | 1 – 50887 | Migrastatin / Dorrigocin (45%) |
| TransAT-PKS-like, Butyrolactone | 89 | 1 – 19037 |  |
| *AT - acetyltransferase. NRPS - nonribosomal peptide synthetase. PKS - polyketide synthase. RiPP - ribosomally synthesized and post-translationally modified peptide.* | | | |

**Supplementary Table 4.** Summary of Tukey post hoc Tests on BGC abundance in *Massilia* genomes.

|  | **Comparison** | **Estimated Difference** | **Std. Error** | **t value** | **Pr(>\|t\|)** |
| --- | --- | --- | --- | --- | --- |
| Raw abundance | Arctic/alpine to Antarctic | -6.643 | 1.541 | -4.31 | < 0.001 |
|  | Mesophile to Antarctic | -6.318 | 1.276 | -4.95 | < 0.001 |
|  | Mesophile to Arctic/alpine | 0.325 | 1.202 | 0.27 | 0.960 |
|  |  |  |  |  |  |
| Normalized to genome size*10^6^ | Arctic/alpine to Antarctic | -0.495 | 0.201 | -2.467 | 0.048 |
|  | Mesophile to Antarctic | -0.464 | 0.166 | -2.793 | 0.023 |
|  | Mesophile to Arctic/alpine | 0.031 | 0.156 | 0.198 | 0.978 |
|  |  |  |  |  |  |
| Normalized to number of coding sequences*10^3^ | Arctic/alpine to Antarctic | -0.598 | 0.236 | -2.536 | 0.041 |
|  | Mesophile to Antarctic | -0.548 | 0.195 | -2.808 | 0.021 |
|  | Mesophile to Arctic/alpine | 0.050 | 0.184 | 0.272 | 0.955 |

**Supplementary Table 5.** Table of prodiginine features detected by MPACT **(Samples et al., 2022)**.

| **Compound** | ***m/z*** | **Retention Time (min)** | **FC** | **max** | **-logp** | **-logq** | **logFC** |
| --- | --- | --- | --- | --- | --- | --- | --- |
| 6.883_266.12851 | 266.12851 | 6.883 | 0.338 | 2.62E+07 | 4.97 | 4.62 | -1.57 |
| 6.894_282.15964 | 282.15964 | 6.894 | 0.625 | 4.34E+07 | 3.68 | 3.52 | -0.68 |
| 5.963_294.1597 | 294.1597 | 5.963 | 2.167 | 2.67E+07 | 2.12 | 2.04 | 1.12 |
| 7.328_296.17532 | 296.17532 | 7.328 | 0.010 | 1.39E+07 | 6.35 | 5.76 | -6.64 |
| 7.328_298.19122 | 298.19122 | 7.328 | 0.010 | 1.84E+07 | 5.80 | 5.32 | -6.64 |
| 6.88_308.17499 | 308.17499 | 6.880 | 0.335 | 1.58E+07 | 3.80 | 3.63 | -1.58 |
| 7.328_308.17569 | 308.17569 | 7.328 | 0.014 | 2.74E+07 | 5.64 | 5.18 | -6.20 |
| 6.739_310.19122 | 310.19122 | 6.739 | 0.265 | 2.48E+07 | 0.98 | 0.94 | -1.91 |
| 7.325_310.1915 | 310.1915 | 7.325 | 0.010 | 2.37E+09 | 4.02 | 3.79 | -6.64 |
| 6.886_322.19125 | 322.19125 | 6.886 | 0.328 | 2.06E+07 | 4.60 | 4.30 | -1.61 |
| 6.635_322.19153 | 322.19153 | 6.635 | 0.097 | 1.06E+07 | 2.15 | 2.06 | -3.37 |
| 6.881_324.20724 | 324.20724 | 6.881 | 0.083 | 8.89E+09 | 3.59 | 3.44 | -3.58 |
| 7.326_326.18634 | 326.18634 | 7.326 | 0.017 | 2.36E+09 | 6.04 | 5.51 | -5.87 |
| 6.992_326.1864 | 326.1864 | 6.992 | 0.010 | 2.74E+08 | 5.39 | 5.00 | -6.64 |
| 6.746_326.18655 | 326.18655 | 6.746 | 0.010 | 5.62E+08 | 5.99 | 5.47 | -6.64 |
| 5.977_326.18665 | 326.18665 | 5.977 | 0.016 | 2.26E+08 | 2.86 | 2.74 | -6.01 |
| 6.893_340.20172 | 340.20172 | 6.893 | 0.601 | 1.33E+09 | 3.78 | 3.61 | -0.73 |
| 6.461_340.20197 | 340.20197 | 6.461 | 5.475 | 4.77E+07 | 5.44 | 5.03 | 2.45 |
| 6.521_340.20209 | 340.20209 | 6.521 | 0.407 | 3.61E+07 | 1.56 | 1.51 | -1.30 |
| 6.146_340.20221 | 340.20221 | 6.146 | 1.837 | 2.18E+07 | 1.84 | 1.77 | 0.88 |
| 7.317_352.23792 | 352.23792 | 7.317 | 0.013 | 1.10E+08 | 7.44 | 6.38 | -6.22 |
| 7.265_352.23822 | 352.23822 | 7.265 | 0.039 | 7.42E+07 | 7.57 | 6.41 | -4.70 |
| 7.854_354.21735 | 354.21735 | 7.854 | 0.010 | 6.42E+07 | 3.61 | 3.46 | -6.64 |
| 7.088_354.21753 | 354.21753 | 7.088 | 0.245 | 5.82E+07 | 3.00 | 2.87 | -2.03 |
| 7.275_368.23285 | 368.23285 | 7.275 | 0.116 | 3.53E+08 | 6.06 | 5.51 | -3.11 |
